# Modelling speed behaviour in rural highways: Safety analysis of driving under adverse road-weather conditions

**DOI:** 10.1371/journal.pone.0256322

**Published:** 2021-08-16

**Authors:** Rillagoda G. N. Yasanthi, Babak Mehran, Wael K. M. Alhajyaseen

**Affiliations:** 1 Department of Civil Engineering, University of Manitoba, Winnipeg, Manitoba, Canada; 2 Qatar Transportation and Traffic Safety Center, Qatar University, Doha, Qatar; Tongii University, CHINA

## Abstract

This study proposes a methodical approach to model desired speed distributions under different road-weather and traffic conditions followed by identification of road-weather conditions with potentially higher safety risks in rural divided highways located in extremely cold regions. Desired speed distributions encompassing unique combinations of adverse road-weather and traffic conditions are modelled as normal distributions characterized by their means and standard deviations formulated based on two principal statistical theorems and techniques i.e., Central Limit Theorem and Minimum Variance Unbiased Estimation. Combination of the precipitation conditions, road surface conditions, time of the day, temperature, traffic flow and the heavy vehicle percentage at the time of travel were considered in defining the combinations of road-weather and traffic conditions. The findings reveal that simultaneous occurrence of particular precipitation and pavement conditions significantly affect the characteristics of the desired speed distribution and potentially expose drivers to elevated safety risks. Jurisdictions experiencing extreme road-weather conditions may adapt the proposed methodology to assess speed behaviour under different road-weather conditions to establishing and deploying weather-responsive traffic management strategies such as variable speed limit to regulate speeding and improve traffic safety in winter.

## Introduction

Highway safety, characterized by the ability of a person to travel freely without injury or death, has always been the primary objective of traffic engineering and is typically measured by the rate of crashes belonging to different severity levels [1]. As defined by [2], a cause of a crash can be defined as “a circumstance or an action that, were it different, the frequency of crashes and/or their severity would be different”. The contributing factors to crashes can be divided into four major categories: human factors (driver, pedestrian, etc. behavior), vehicle conditions, roadway conditions and environmental conditions [3]. A significant proportion of crashes includes weather-related crashes, which are defined as crashes occurring in the presence of rain, sleet, snow, fog, wet pavement, snowy/slushy pavement, and/or icy pavement [4]. In fact, [5] identified 307 fatalities resulted from weather-related crashes in Canada in 2016. It is well documented in the literature that weather-related crashes mostly stem from the impacts of adverse road-weather conditions on driver behaviour [4, 6].

Speed is considered as one of the major contributing factors to crashes in terms of frequency and severity, thus it has been widely used as a surrogate measure in assessing highway safety [7]. Speed limits posted by jurisdictions regulate and influence drivers’ speed choice and attempts to mitigate crash risks [8]. It is hypothesized that particular road-weather conditions may alter drivers’ speed choice and intensify crash risks. Therefore, fixed speed limits may not effectively mitigate crash risks under adverse road-weather conditions. Variable Speed Limit (VSL) which is implemented for real-time crash mitigation [7] is a viable weather-responsive traffic management strategy to alleviate safety risks imposed by adverse road-weather conditions. Thus, understanding the speeding behaviour under adverse road-weather conditions is essential for successful implementation of a weather-responsive VSL program. Desired speed, which is the speed chosen at driver’s discretion at specific traffic and roadway conditions directly reflects driver behaviour and is extensively used in studying driver behaviour [9, 10]. Desired speed is manifested through the free-flow speed of vehicles [11] and is referred to as “speed” hereafter in. In fact, [11] recommends taking the speed observed under base conditions at traffic flow volumes less than 1,000 passenger cars per hour per lane, as a reliable measure of free-flow speed as traffic becomes insensitive to flow rate at this much low traffic volumes. Distribution of desired speeds at a specific location varies in a wide range mainly attributing to geometric characteristics of the road, existing road-weather conditions and the demographics of the drivers. In other words, desired speed explains the speed at which the drivers are comfortable under the prevalent driving conditions. Adverse road-weather conditions such as icy road surface conditions and poor visibility are proven to affect the desired speed significantly [9, 12]. Accordingly, speed behavior, which is defined as the choice between several possible speeds [13], is widely chosen as a measure to study the impacts of adverse road-weather conditions on driver behaviour.

Past literature explored several approaches to investigate the impacts of adverse road-weather conditions on speed behaviour including statistical modelling and machine learning techniques [9, 14, 15]. For instance, [9] developed a group of statistical models to enumerate the speed variations resulted by adverse road-weather conditions, where the set of multiple linear regression models with 20-minute aggregate speeds as the dependent variable (Group II models) was concluded as the best performing model. Hoogendoorn [16] developed a new unified approach to estimate the free speed distributions based on the composite headway distribution model and the method of censored observations.

While some studies confirmed significant changes in speed behaviour under adverse road-weather conditions [12, 14, 17], other studies contradict these observations, mainly in terms of the intensity of speed dispersion [9, 18]. Further, the performance of conventional approaches such as regression modelling to study speed behaviour has been criticized [9]. The underlying reasons for discrepancies in the literature are mainly threefold. The main prospective reason discussed is limited sampling to represent specific road-weather conditions [9, 19]. On one hand, adverse road-weather conditions are intrinsically scarce, restricting the number of samples, which can be collected under specific inclement road-weather conditions. On the other hand, the travel patterns are significantly affected by adverse road-weather conditions resulting in reduced traffic volumes under adverse road-weather conditions [20]. Limited sample rate under adverse road-weather conditions results in inconsistent speed reduction estimations and limits the applicability of desired speed distribution modelling approaches developed by previous studies. For instance, free speed distribution models developed in [10] and [16] require sufficiently large samples to minimize the standard error of the estimates, which may not be feasible under adverse road-weather conditions. Second, most of the studies relied on road-weather data collected from data collection devices located significantly distant from the traffic data collection stations, which potentially reduces the representativeness of prevailing road-weather conditions of the collected data. Third, treatment of speed data (e.g. aggregation interval) also appears to impact the outcomes [9, 12]. While it is common to analyze speed behaviour through statistical modelling, it should be noted that speed behavior is primarily a human response. Thus, modelling human psychology through regression modelling, especially with a limited number of samples is challenging and may lead to questionable results [21]. Further, the form of the dependent variables in the statistical models, whether aggregated or disaggregated as well as the aggregation interval is proven to impact the study results [9]. In fact, the optimum aggregation interval for loop detector data (i.e., speed data) depends on the purpose of the application and traffic conditions [22]. Moreover, many researchers queried about the functional form to be used in modelling the impacts of road-weather conditions on traffic stream characteristics of uninterrupted flow [9, 14].

While using identical study data as used by [9], this study intends to fill the gaps identified in the literature by proposing a novel approach for modeling the distribution of desired speeds in uncongested highways under different combinations of road-weather and traffic conditions, rather than quantifying speed variations resulted by adverse road-weather conditions. The proposed methodology embodies two novelties. First, it attempts to identify the impacts of adverse road-weather conditions on drivers’ desired speed by modelling desired speed distributions, rather than quantifying the speed variations resulted by adverse road-weather conditions through statistical modelling (e.g., regression analysis), thus avoiding well-documented issues such as poor goodness of fit and insignificant model coefficients which are attributed to the low sample sizes and the divergent nature of human behaviour. We attempt to resemble the speed choice of a specific driver population rather than attempting to confine drivers’ speed behavior by a specific mathematical criterion such as ordinary least squares method. Accordingly, the proposed methodology can be practiced under limited sampling conditions as it preserves all observations. Second, we attempt to evaluate the impacts of different road-weather conditions on a population-level in contrast to the conventional sample-level evaluations. One of the main advantages of the proposed novel approach is that modelling the desired speed distributions eliminates the need to define a specific function or an aggregation interval. Moreover, road-weather and traffic data collection devices used in this study are located alongside each other providing highly accurate data.

## Study objectives

This study attempts to address two research questions related to rural divided highways: *i*) How does the distribution of desired speeds change in the presence of adverse road-weather conditions? and *ii*) Are there specific adverse road-weather conditions, which significantly intensify safety risks?

The study has two objectives focusing on addressing the research questions:

- To model the desired speed distributions under the combined effect of specific road-weather and traffic conditions, and
- To identify specific adverse road-weather conditions which significantly intensify the safety risks.

The distribution of traffic speeds is primarily dependent on drivers’ speed choice ranging from very low to very high speeds. According to Hauer [23], slow drivers habitually select lower speeds because they believe that slow driving is safe driving. On the other hand, fast drivers trade off safety for lesser travel times [23]. In this study, it is hypothesized that the population of drivers under specific road-weather conditions choose a safe speed to travel based on their comfort, convenience and confidence to travel under prevailing road-weather conditions. Further, it is hypothesized that the divergent speed selection patterns under specific adverse road-weather conditions increase the variability of desired speeds, which is an indication of increased safety risks. The study attempts to test these hypotheses by estimating desired speed distribution parameters for different populations of drivers under different road-weather and traffic conditions. The study results will help authorities establish weather-responsive traffic management schemes i.e., VSL to improve traffic safety and operations under adverse road-weather conditions in cold regions.

## Study data

### Data collection

**Fig 1:**
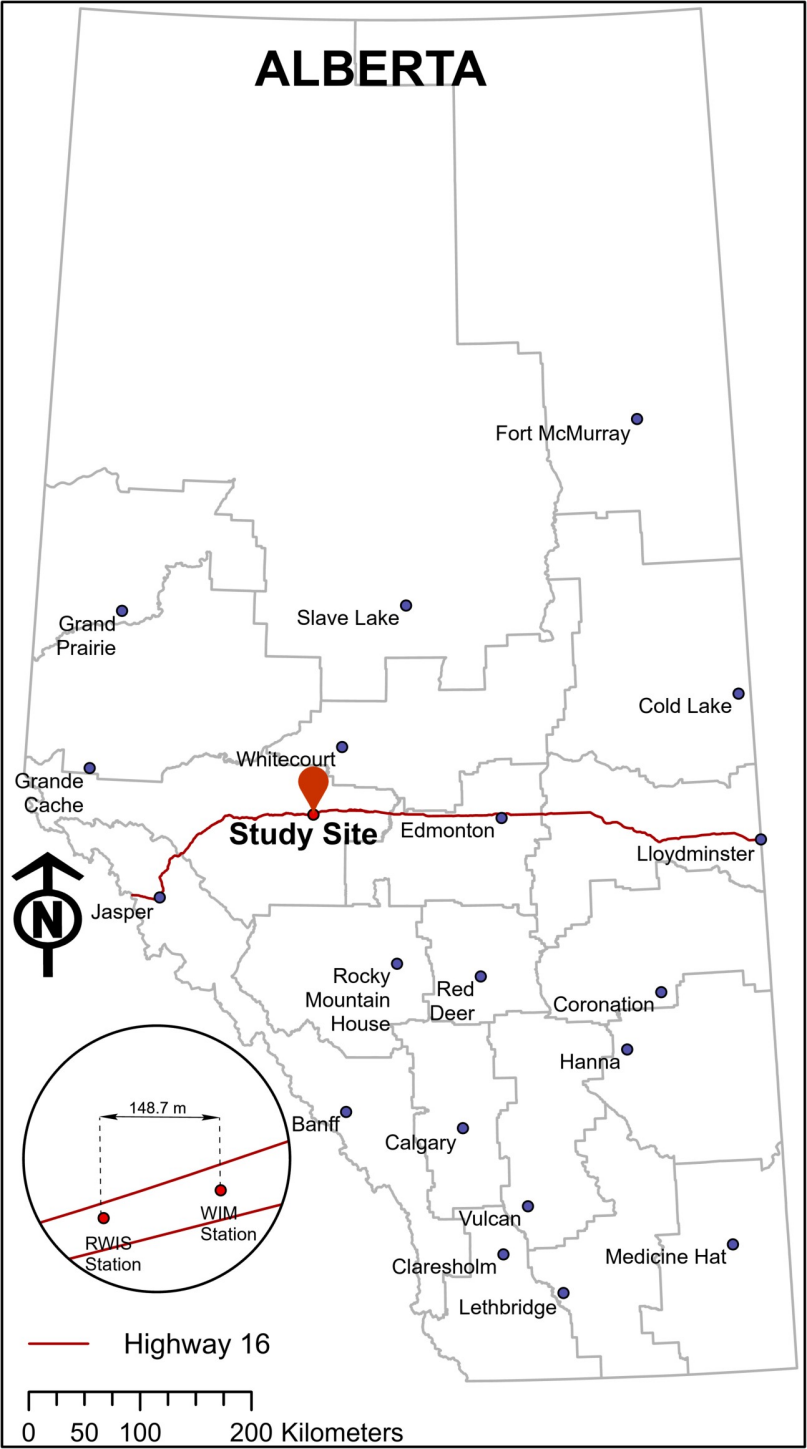
Study site location and the positions of the RWIS & the WIM station (map generated using the data retrieved from [25–27] and contains information licensed under the Open Government Licence-Canada).

The study data were collected with the courtesy of Alberta Transportation and are of two types; road-weather and traffic data. Road-weather data were collected by using a Road Weather Information System (RWIS) and traffic data were acquired by a Weigh-In-Motion (WIM) station installed alongside of each other (148.7m apart) on a four-lane, two-way divided highway segment (Fig 1). The study site is located on Highway 16 i.e., a major inter-provincial highway in Western Canada a.k.a “Yellowhead highway”. It connects Jasper and Lloydminister via Edmonton, and the study site lies west to the city of Edmonton. The location configuration of RWIS and WIM sensors (Fig 1) enabled collecting real-time, highly accurate and representative road-weather information for each vehicle recorded by the WIM station, adding a distinct feature to this study. The study site was subjected to an Annual Average Daily Traffic (AADT) of 8,120 vehicles in 2015 [24]. Moreover, the study site is in a level, straight road section without any on/off ramps nearby. The study data were collected for 15 months ranging from October 2014 to December 2015.

The RWIS station records the pertaining road-weather conditions including air temperature, atmospheric precipitation situation, pavement surface condition, pavement temperature, and wind average speed in every 20 minutes. In terms of traffic data, the WIM station detects the date, time and vehicle-by-vehicle information including travel lane, travel speed, axle weight and interaxial spacing.

### Data preparation

Table 1 summarizes the details of the road-weather conditions as recorded by the RWIS station and the alterations made to the raw data recorded by the RWIS station, in terms of the categorization of continuous data and/or re-grouping of the categorical data records. Initially, atmospheric temperature, precipitation situation, pavement surface condition, wind speed and time of the day were selected as independent variables representing road-weather conditions as discussed in [9]. However, wind speed was not considered in the analysis since the maximum wind speed recorded at the study site is 45.36 km/h, which is lower than the reported minimum wind speed required (51 km/h) to impact the behavior of passenger car drivers [28]. A Kolmogorov–Smirnov (K-S) test was conducted to assess the statistical significance of each level in the remaining categorical road-weather attributes. Consequently, the following alterations were executed:

- Original precipitation conditions recorded by the RWIS station were combined in terms of the intensities due to the statistically insignificant difference among the “Moderate” and “Heavy” levels in each precipitation condition (Table 1). The resultant precipitation conditions only have two levels labeled “slight” and “Moderate & Heavy” each for rain, frozen precipitation and snow.
- Air temperature was categorized into three groups (GI, GII and GIII) based on the numerical value of the air temperature as shown in Table 1.
- Precipitation condition, pavement surface condition, temperature and time of the day were modified to have seven, six, three and two levels, respectively. Levels of a particular road-weather category were subjected to a K-S test to confirm the statistical independence of each level from the remaining groups for the specific Road-Weather (RW) variable in question.

**Table 1:** Road-weather condition categories.

RW^1^ condition	Description of the raw data as recorded by RWIS			Alterations to the raw data
Air temperature	Wet bulb temperature falling between minus and plus 40 recorded as a continuous variable			GI: Temperature ≤−10°C
				GII: −10°C<Temperature ≤ 0°C
				GIII: Temperature >0°C
Pavement surface condition	Dry	No moisture or unusual condition detected		No alterations
	Ice warning	Detection of ice or black ice		
	Trace moisture	Detection of isolated moisture on pavement surface		
	Wet	Wet roadway with significant moisture detection		
	Ice watch	The risk of the formation of ice or black ice on the roadway is elevated, but its occurrence, location, and/or timing is still uncertain		
	Frost	Detection of frost formation		
Time of the day	Day	Time from the sunrise to sunset		No alterations
	Night	Time from the sunset to sunrise		
Precipitation condition	No precipitation	0 mm/h	No precipitation	No alterations
	Rain	<2 mm/h	Slight rain	No alterations
		≥2 and <8 mm/h	Moderate rain	Moderate & heavy rain
		≥8 mm/h	Heavy rain	
	Snow	<2 mm/h	Slight snow	No alterations
Precipitation condition	Snow	≥2 and <8 mm/h	Moderate snow	Moderate & heavy snow
		≥8 mm/h	Heavy snow	
	Frozen precipitation	<2 mm/h	Slight frozen precipitation	No alterations
		≥2 and <8 mm/h	Moderate frozen precipitation	Moderate & heavy frozen precipitation
		≥8 mm/h	Heavy frozen precipitation	Moderate & Heavy Frozen Precipitation

^1^ Road-weather.

As for traffic data, erroneous records including “Error”, “Other” and “Not Applicable” entries as well as vehicles with speeds higher than 200km/h were removed from the analysis. Further, the study data were aggregated into five-minute intervals, which is suggested as the optimum interval to investigate traffic operations [29]. Consequently, traffic flow and the percentage of heavy vehicles for each five-minute interval count were estimated. Traffic flow was grouped into eight levels with a bin size of 100 veh/h. Likewise, heavy vehicle percentage was grouped into 10 levels with a bin size of 10%. Traffic flow and heavy vehicles percentage categories were also subjected to K-S test to verify their statistical significance. Accordingly, each five-minute interval during the analysis period was tagged with the prevailing precipitation, pavement condition, temperature, time of the day, heavy vehicle percentage and traffic flow categories at the time of travel.

Table 2 tabulates statistical information of the influencing factors with respect to several statistical attributes; number of vehicles recorded under each level of the influence factor along with the mean and standard deviation where applicable.

**Table 2:** Summary statistics of influencing factors.

Influencing factor			Vehicles observed	Mean	Standard deviation
Road-weather conditions	Air temperature	GI (≤ −10°C)	230,489	-16.58°C	4.77°C
		GII (−10°C<&≤ 0°C)	441,030	-3.98°C	2.79°C
		GIII (>0°C)	880,823	8.74°C	6.35°C
	Pavement surface condition	Dry	958,017	Not Applicable	
		Ice warning	269,603	Not Applicable	
		Trace moisture	41,498	Not Applicable	
		Wet	134,556	Not Applicable	
		Ice watch	132,540	Not Applicable	
		Frost	16,128	Not Applicable	
	Time of the day	Day	1,159,360	Not Applicable	
		Night	392,982	Not Applicable	
	Precipitation condition	No precipitation	1,448,239	Not Applicable	
		Slight rain	21,429	0.48 mm/h	0.54 mm/h
		Moderate & heavy rain	46,397	6.6 mm/h	4.29 mm/h
		Slight snow	23,332	0.55 mm/h	0.47 mm/h
		Moderate & heavy snow	1,685	2.96 mm/h	2.73 mm/h
		Slight frozen precipitation	9,578	0.63 mm/h	0.75 mm/h
		Moderate & heavy frozen precipitation	1,682	5.28 mm/h	5.27 mm/h
Traffic conditions	Traffic flow category	TF: GI (≤100 veh/h)	38,923	48 veh/h	27 veh/h
		TF: GII (101 veh/h-200 veh/h)	26,544	152 veh/h	28 veh/h
		TF: GIII (201 veh/h-300 veh/h)	27,953	248 veh/h	31 veh/h
		TF: GIV (301 veh/h-400 veh/h)	10,962	346 veh/h	27 veh/h
		TF: GV (401 veh/h-500 veh/h)	3,107	440 veh/h	27 veh/h
		TF: GVI (501 veh/h-600 veh/h)	767	538 veh/h	29 veh/h
		TF: GVII (601 veh/h-700 veh/h)	170	646 veh/h	29 veh/h
		TF: GVIII (701 veh/h-800 veh/h)	94	752 veh/h	30 veh/h
	Heavy vehicles percentage category	HV: GI (≤ 10%)	19,492	3.1%	3.8%
		HV: GII (11%-20%)	20,410	15.8%	3.0%
		HV: GIII (21%-30%)	19,032	25.4%	2.7%
		HV: GIV (31%-40%)	16,847	35.2%	23.0%
		HV: GV (41%-50%)	13,623	47.5%	3.1%
		HV: GVI (51%-60%)	3,919	57.2%	2.5%
		HV: GVII (61%-70%)	4,275	66.1%	1.7%
		HV: GVIII (71%-80%)	2,656	75.8%	2.7%
		HV: GIX (81%-90%)	576	84.6%	1.9%
		HV: GX (>90%)	7,690	99.9%	0.3%

#### Preliminary analysis

A preliminary data analysis was conducted on the eastbound data (a similar analysis could be conducted on westbound data) aiming to: *i*) identify the level of vehicle interactions at the study site, *ii*) understand the overall speed behaviour in different road-weather conditions, and *iii*) analyze traffic composition and lane utilization patterns at the study site.

**Fig 2:**
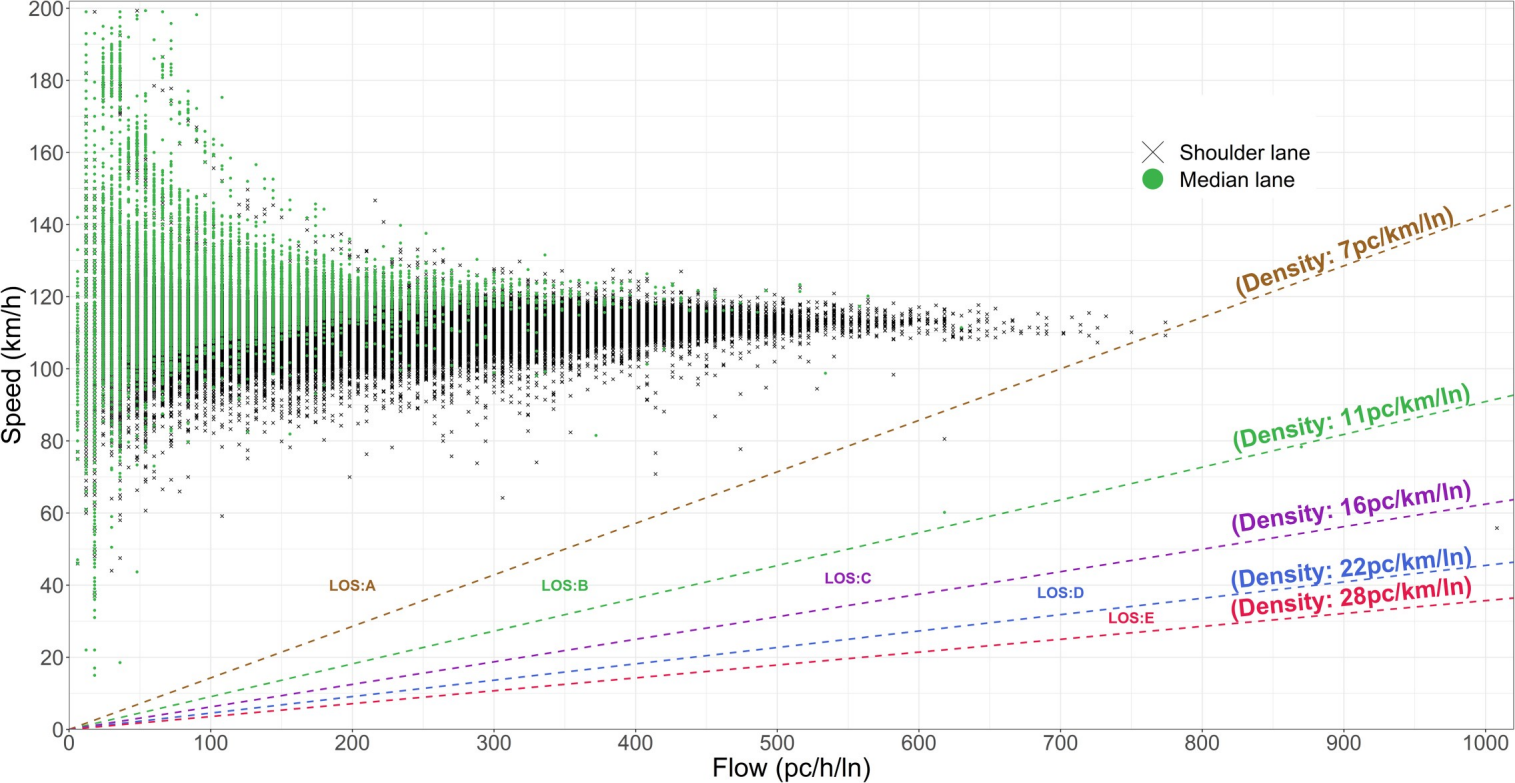
Speed-flow variation in the eastbound shoulder and median lanes at the study site.

Fig 2 depicts the speed-flow relationship in the eastbound shoulder and median lanes separately, where flow and speed are expressed in the hourly equivalent of the five-minute passenger car volumes per lane and the corresponding five-minute aggregate speed respectively. According to Fig 2, the study site mostly operates under a density of 7pc/h/ln and experiences Level of Service (LOS) “A”, which exhibits free-flow conditions and minimal vehicle interactions between the vehicles as characterized by [29].

**Fig 3:**
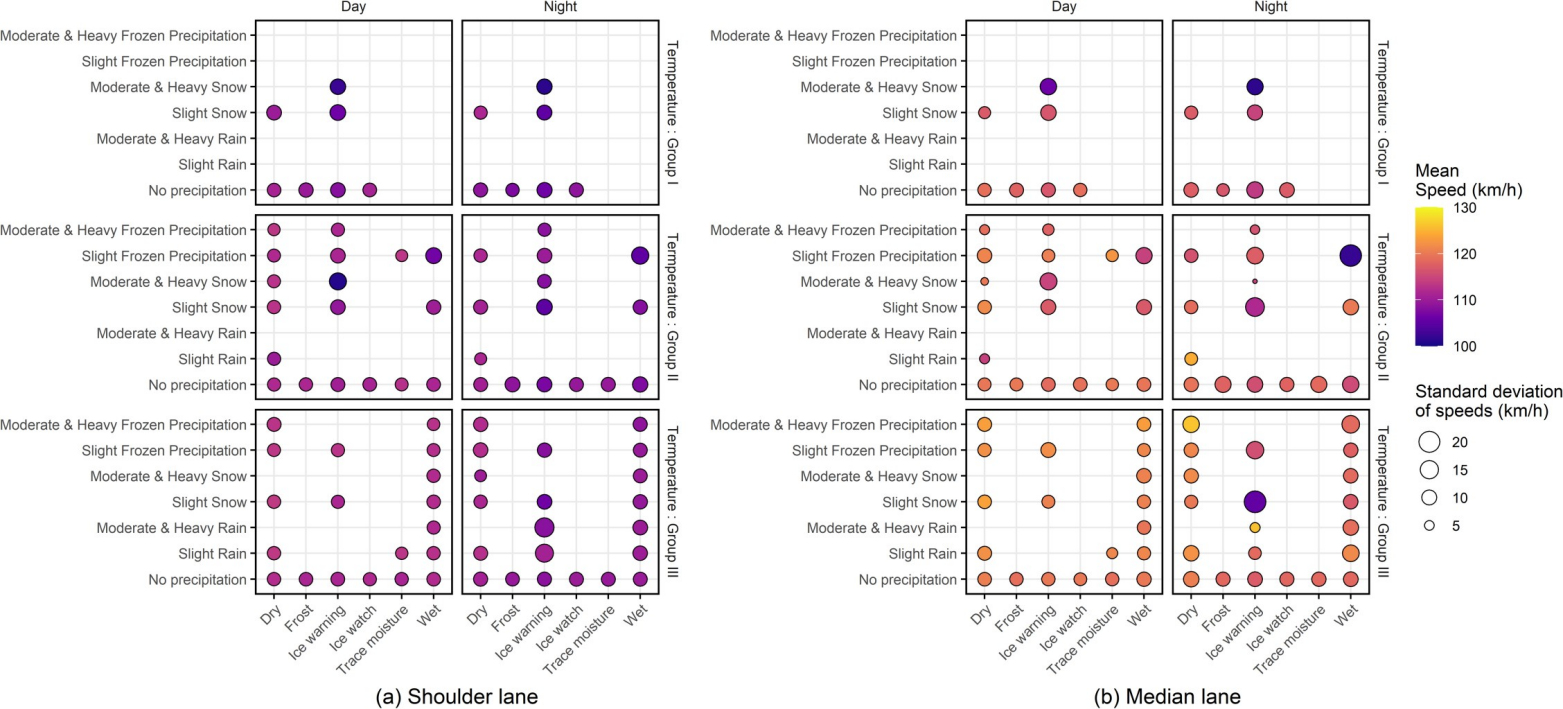
Distribution of speeds in the study site.

The median lane, however, exhibits a lower range of traffic flow values and higher speeds compared to the shoulder lane. While presenting two types of descriptive statistics; mean and standard deviation of the speeds observed in the eastbound shoulder and median lanes, Fig 3 confirms observation of higher speeds in the median lane while highlighting the variations in the mean and standard deviation of speeds under adverse road-weather conditions in both lanes.

Further, vehicles travelling in daytime, especially in the shoulder lane travel at slightly higher speeds. Adverse pavement surface conditions, when coupled with adverse precipitation conditions tend to reduce speeds significantly. For instance, presence of an ice warning pavement condition under a slight snow recorded significantly lower mean speeds and considerably high standard deviation of speeds irrespective of the temperature conditions, time of day and the lane type, suggesting that the drivers are more sensitive to perceptible hazards such as adverse pavement and precipitation conditions. Fig 3 suggests a few counterintuitive implications potentially due to the small sample sizes. For example, the mean and standard deviation of speeds of vehicles travelling in the median lane at nighttime in temperature values more than 0°C under a dry pavement and a moderate and heavy frozen precipitation recorded a comparatively higher mean speed which can potentially be attributed to the low sample size (44 five-minute intervals only).

**Fig 4:**
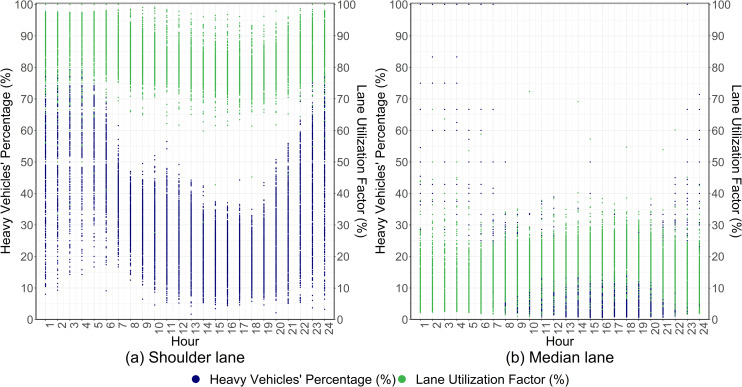
Hourly variation of the heavy vehicles’ percentage and the lane utilization factor at the study site.

Fig 4 presents the hourly variation of the heavy vehicles’ percentage and the lane utilization factor in the eastbound shoulder and median lanes, where the lane utilization factor is expressed in terms of the proportion of hourly vehicular flow of each lane to the respective total hourly vehicular flow of the eastbound direction. According to Fig 4, a substantial portion of the hourly vehicular flow in the shoulder lane constitutes of heavy vehicles, especially around the midnight. Moreover, the high hourly lane utilization factors of the shoulder lane further imply the preference of the majority of the drivers to use the shoulder lane. Therefore, only the vehicles observed in the shoulder lane are considered in this study to typify the pragmatic heterogeneous traffic composition in study site.

### Population, sample and sampling distributions

It is hypothesized that drivers possess different speed behaviors under various inclement road-weather and traffic conditions, characterized by a “population” to which they belong to. Three important aspects in modelling the distribution of desired speeds are defined namely, population, sample and sampling distribution.

**Fig 5:**
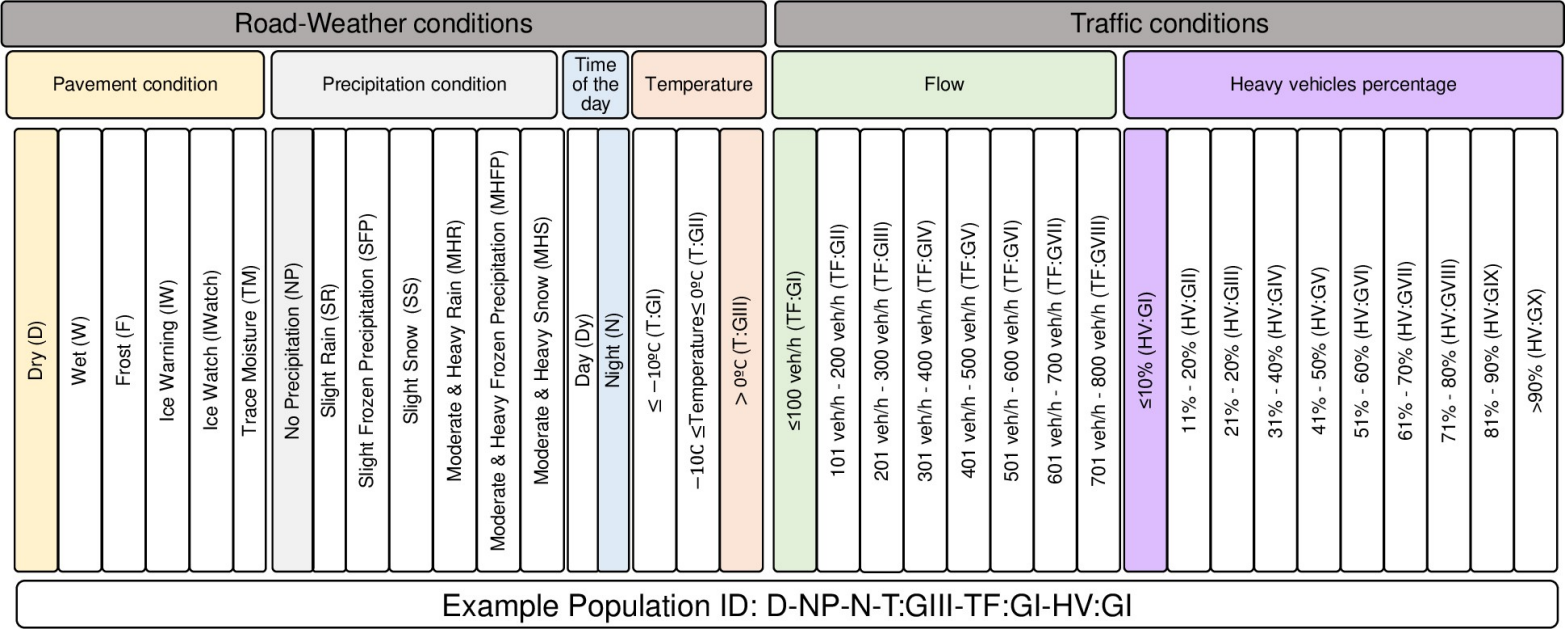
Categories of road-weather and traffic conditions to identify populations.

Population, in the context of this study, encompasses the desired speeds of individual vehicles observed at the study site under a specific combination of road-weather and traffic conditions. Population groups are identified based on the combination of six criteria explaining the road-weather and traffic conditions at the time of travel (Fig 5). The distribution of desired speeds of the individual vehicles observed under a unique combination of road-weather and traffic conditions is referred to as “population distribution” hereinafter.

The study period is divided into consecutive five-minute intervals analogous to samples drawn from the speed population in equal intervals. Each five-minute interval belongs to a specific population labeled with a proper population ID based on the prevailing road-weather and traffic conditions. The individual speeds in each time interval are considered as “observations” while the respective five-minute intervals are considered as “samples” drawn from a population. Accordingly, the number of vehicles observed in a particular five-minute interval is referred to as the “sample size” denoted by *n* (*n*∈ℤ^+^) i.e., the five-minute traffic volume. The number of unique sample sizes that belong to a particular population is denoted by *k* (*k*∈ℤ^+^). The average speed of all vehicles observed during a five-minute interval represents the sample mean and is referred to as five-minute aggregate speed. For a particular population, the sampling distribution of mean speed refers to the distribution of the sample mean speeds with a specific sample size *n*. Thus, each speed population is represented by *k* sampling distributions.

**Fig 6:**
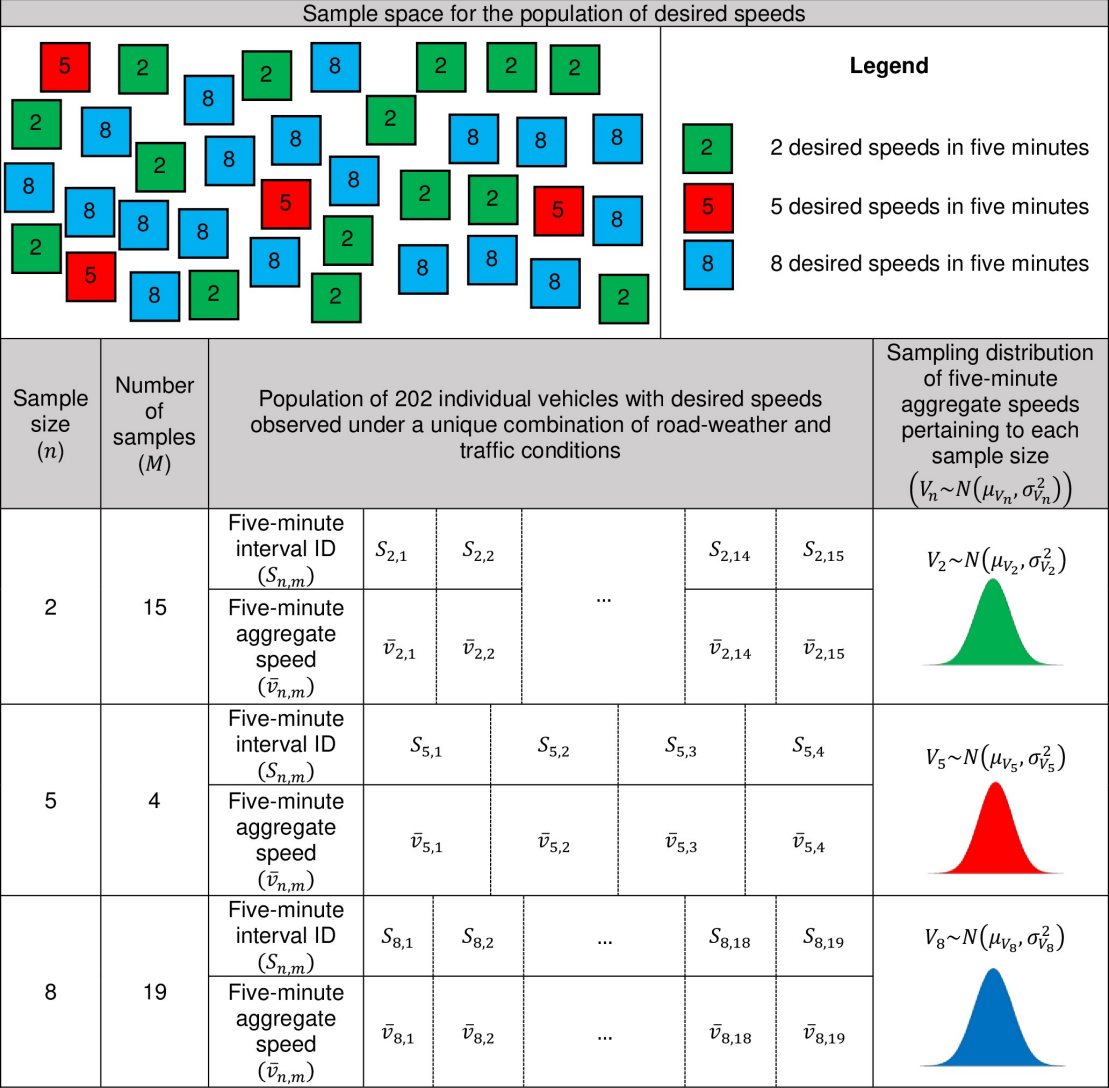
Illustration of a population and samples.

For demonstration purpose, Fig 6 presents a graphical illustration of a speed population of 202 individual vehicle speeds recorded under a unique combination of road-weather and traffic conditions, enclosed in 38 five-minute intervals belonging to three unique sample sizes (*k* = 3). Each five-minute aggregate interval in the population is assigned with a unique sample ID denoted by *S*_*n*,*m*_, where *n* denotes the sample size and *m* (1≤*m*≤*M*) denotes the sample number pertaining to the sample size *n*. The number of five-minute intervals recorded with identical five-minute volumes is denoted by *M* (*M*∈ℤ^+^). The five-minute aggregate speed in each five-minute interval represents the sample mean speed and is denoted by ${\bar{v}}_{n,m}$. The five-minute aggregate intervals observed with identical number of vehicles are clustered together to produce sampling distributions of mean speed. Accordingly, *V*_*n*_ represents the sampling distribution of five-minute aggregate speeds pertaining to intervals with traffic volume *n*. Five-minute intervals with *M* = 1 were removed and the rest of the samples are sorted in an ascending order based on *n*.

**Fig 7:**
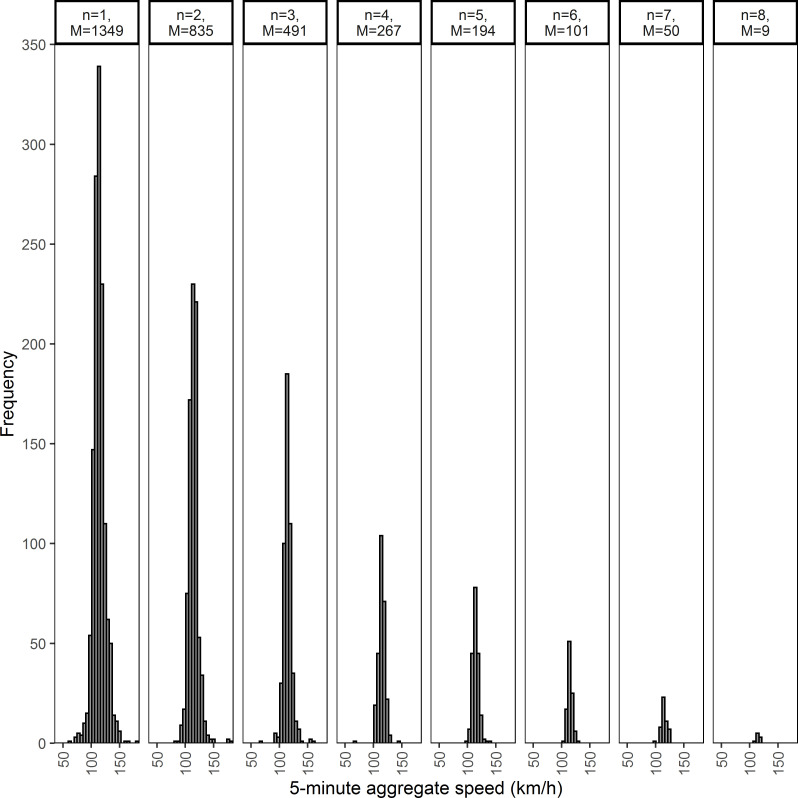
Sampling distribution of mean speeds for a population represented by eight sampling distributions (population ID: D-NP-N-T:GIII-TF:GI-HV:GI).

Fig 7 shows an example of a speed population (ID: D-NP-N-T:GIII-TF:GI-HV:GI) i.e. dry pavement, no precipitation, temperature more than 0°C, q<100 veh/h and a HV<10% in nighttime) represented by 8 different sampling distributions of speeds *(k = 8)* along with the respective number of five-minute intervals (*M*). For instance, the fourth sampling distribution (*n =* 4, *M* = 267) in Fig 7 corresponds to the distribution of the five-minute aggregate speeds encompassing 267 five-minute intervals with four vehicles observed in each five-minutes. The frequency of samples for each sample size *n* tends to decrease as the sample size increases implying comparatively low traffic volumes observed at the study site, which is in an uncongested rural highway.

## Methodology

The proposed methodology to estimate the mean and standard deviation of the distribution of desired speeds of individual vehicles is founded upon two main assumptions; *i)* observed speeds on the study segment represent drivers’ desired speed, and *ii)* the population of observed individual vehicle speeds belonging to a unique combination of traffic and road-weather conditions is normally distributed and is represented by a mean of *μ* and a standard deviation of *σ*. Nevertheless, the study data were carefully checked to scrutinize the contrarieties between the theoretical postulations and the empirical indications. To implement the proposed methodology, the observed speeds should be first affirmed as desired speeds, characterized as the speeds of lead vehicles i.e., non-followers or observed speed of vehicles with minimal interactions [10]. For congested highways with significant vehicle interactions a “follower/non-follower” identification algorithm such as the composite headway distribution model [16] can be applied to identify “follower” and “non-follower” vehicles. Consequently, the analysis can be conducted using the desired speed observations from non-follower vehicles. Alternatively, uncongested traffic streams operating at Level of Service (LOS) “A” or “B” with traffic flow values less than 1,000 veh/h/lane can be reasonably considered to represent “free-flow” conditions [11], where drivers are free to adopt their desired speeds. The shoulder lane of the highway segment in this study mainly operates at LOS “A” and “B” with traffic flow values less than 1,000 veh/h/lane, implying minimal interactions between vehicles (Fig 2). Thus, observed speeds are assumed to represent desired speeds of drivers due to prevailing free-flow conditions at the study site.

On the other hand, normal distribution is often used to represent desired speeds [30] except for deviations under certain situations such as special site characteristics resulting from road geometry [31] and highly heterogeneous traffic [32]. According to Central Limit Theorem (CLT), *V*_*n*_ (sampling distribution of mean speeds) will be nearly normal regardless of the sample sizes given that the speed populations are normally distributed [33]. Accordingly, the characteristics of the study data substantiate the main assumptions of the study.

### Mean of desired speeds’ distribution (*μ*) of individual vehicles’ population

In this study, we hypothesize that the distribution of desired speeds under a specific combination of road-weather and traffic conditions is represented by a random variable *Y* following a normal distribution with a mean of $\mu_{\bar{y}}$ and a standard deviation of $\sigma_{\bar{y}}$. As indicated by CLT, for a normally distributed population, the population mean is equal to the mean of the sampling distribution even for small sample sizes. However, as each speed population is represented by *k* sampling distributions, we propose a methodology to estimate the mean of the speed population by combining *k* sampling distributions (*k* denotes the number of different distributions of five-minute aggregate speeds with specific traffic volumes (sample sizes), comprising a population). In other words, $Y∼N(\mu_{\bar{y}},{\sigma_{\bar{y}}}^{2})$ is analogous to the ultimate result of the linear combination of *k* independent normal random variables, i.e. *k* number of sampling distributions of five-minute aggregate speeds, where the *i*^th^ independent normal random variable and the associated weight factor are denoted by $V_{i}∼N(\mu_{i},\sigma_{i}^{2})$ and *a*_*i*_ respectively, where *i*∈ℤ^+^|*i*≤*k* [34].

**Theorem**: Linear combination of *k* distributions of five-minute aggregate speeds

If $V_{i}∼N(\mu_{i},\sigma_{i}^{2}),$ 1≤*i*≤*k*, are independent random variables and if *a*_*i*_, 1≤*i*≤*k* are constants, then

$$Y=a_{1}V_{1}+\cdots +a_{i}V_{i}+\cdots +a_{k}V_{k}∼N(\mu_{\bar{y}},{\sigma_{\bar{y}}}^{2})$$ Eq 1

where,

$$\mu_{\bar{y}}=a_{1}\mu_{1}+\cdots +a_{i}\mu_{i}+\cdots +a_{k}\mu_{k}$$ Eq 2

and

$${\sigma_{\bar{y}}}^{2}=a_{1}^{2}\sigma_{1}^{2}+\cdots +a_{i}^{2}\sigma_{i}^{2}+\cdots +a_{k}^{2}\sigma_{k}^{2}$$ Eq 3

The mean of the distribution of individual vehicle speeds observed under a unique combination of road-weather and traffic conditions (analogous to a normally distributed population ~*N*(*μ*, *σ*^2^)), is inferred by $\mu_{\bar{y}}$ i.e., the weighted combination of the mean of the distribution of *k* different five-minute aggregate speeds (sampling distributions) representing the population. Nevertheless, estimating $\mu_{\bar{y}}$ evokes two challenges.

First, the aforementioned Theorem only holds true for mutually independent random variables. In the context of this study, the existence of vehicle interactions violates the condition of the observations being mutually independent random variables. Therefore, the mutual independence of the observations must be confirmed prior to applying the linear combination of the distribution of five-minute aggregate speeds. As explained earlier, this study investigates the speed behavior observed in a rural highway, which mostly encounters traffic flow volumes less than 1,000 passenger cars per hour per lane (Fig 2). Hence, it is reasonable to assume negligible vehicle interactions in this analysis. In mathematical terms, recorded speed observations in five-minute intervals can be considered as independent random variables. Thus, *k* distributions of five-minute aggregate speeds are mutually independent.

Second, estimation of $\mu_{\bar{y}}$ also requires the estimation of the weight factors *a*_*i* = 1 *to k*_ (Eq 2). We propose to adapt the “Minimum Variance Unbiased Estimation” (MVUE) technique to estimate the mean of the weighted combination of the distributions of five-minute aggregate speeds ($\mu_{\bar{y}}$) and the respective weight factors (*a*_*i* = 1 *to k*_) pertaining to *Y* bearing the minimum variance (${\sigma_{\bar{y}}}^{2}$). MVUE represents the unbiased point estimate with a minimum variance of all the possible unbiased point estimates for a particular parameter [34]. Therefore, the MVUE of $\mu_{\bar{y}}$ is represented by the mean of a unique distribution i.e., derived by linearly combining *k* distributions of five-minute aggregate speeds, possessing the minimum variance among all other linear combinations. Accordingly, the precise population mean is epitomized by the MVUE of the population mean. Yet, the widely used methods of estimating the MVUEs such as the Cramér–Rao bound (CRB) directly estimates the MVUE without estimating the weight factors [35]. CRB estimates the theoretical lower bound for the variance of the unbiased estimator and a particular estimate is recognized efficient if the CRB is met by the estimate [35]. Nevertheless, MVUE, which matches the lower bound proposed by the CRB may not exist in certain cases [35]. Therefore, a more efficient and practical alternative approach is proposed to estimate the MVUE and the corresponding weight factors for population mean. The precise value of the MVUE of $\mu_{\bar{y}}$ can be obtained by minimizing the variance of the weighted combination of distributions of five-minute aggregate speeds ${\sigma_{\bar{y}}}^{2}$ as explained below.

### Minimum Variance Unbiased Estimate (MVUE) of $\mu_{\bar{y}}$

In the proposed methodology, a population of individual vehicles with the desired speeds observed under a unique combination of road-weather and traffic conditions is represented by *k* sampling distributions of five-minute aggregate speeds, and *Y* is defined in terms of its mean $\mu_{\bar{y}}$ and variance ${\sigma_{\bar{y}}}^{2}$ as in Eqs 1 through 3, respectively. The weight factors *a*_*i* = 1 *to k*_, represent the contribution of the individual sampling distribution of five-minute aggregate speeds *V*_*i*_ in the resulting population distribution for *Y*. Therefore, the weight factors sum up to 1.


$$\sum_{i=1}^{k}a_{i}=1$$ Eq 4

**Fig 8:**
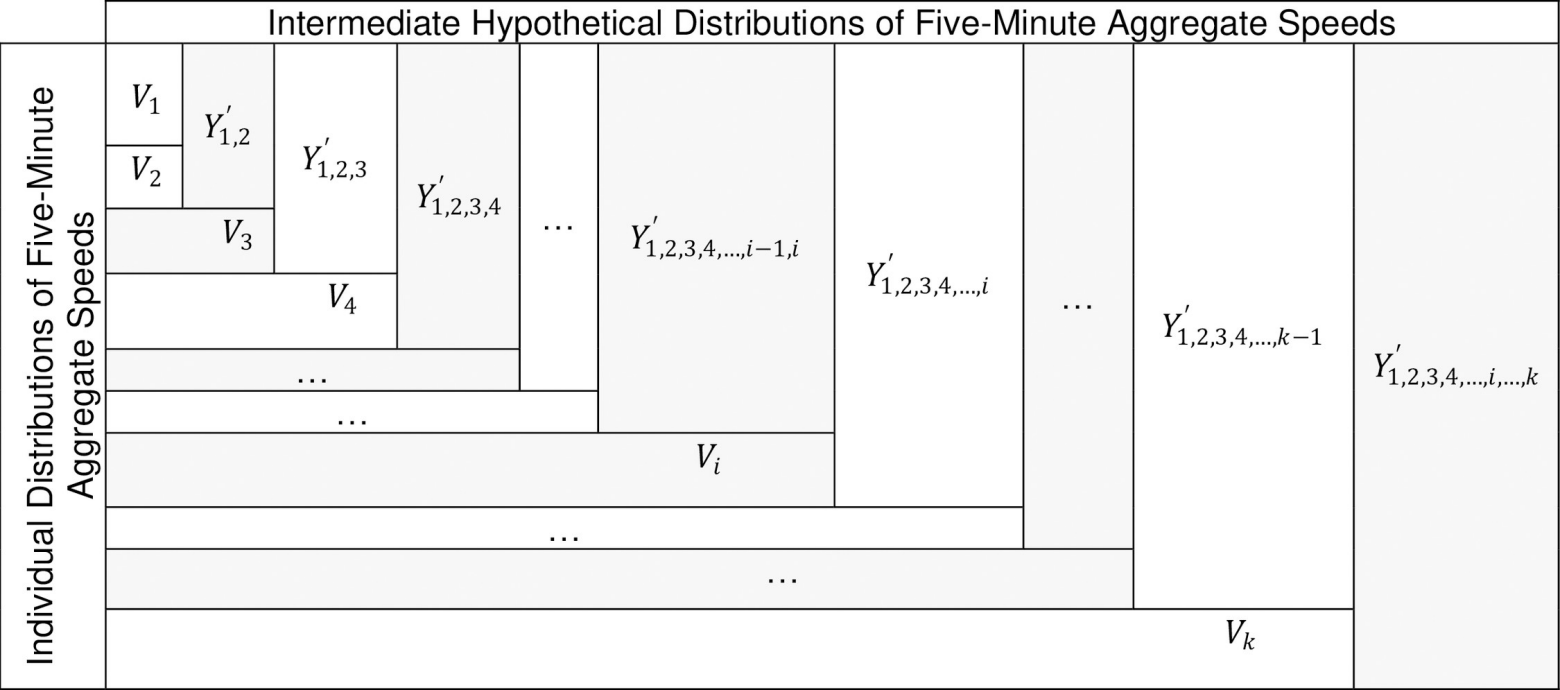
Illustration of the chaining process of k sampling distributions of five-minute aggregate speeds.

**Fig 9:**
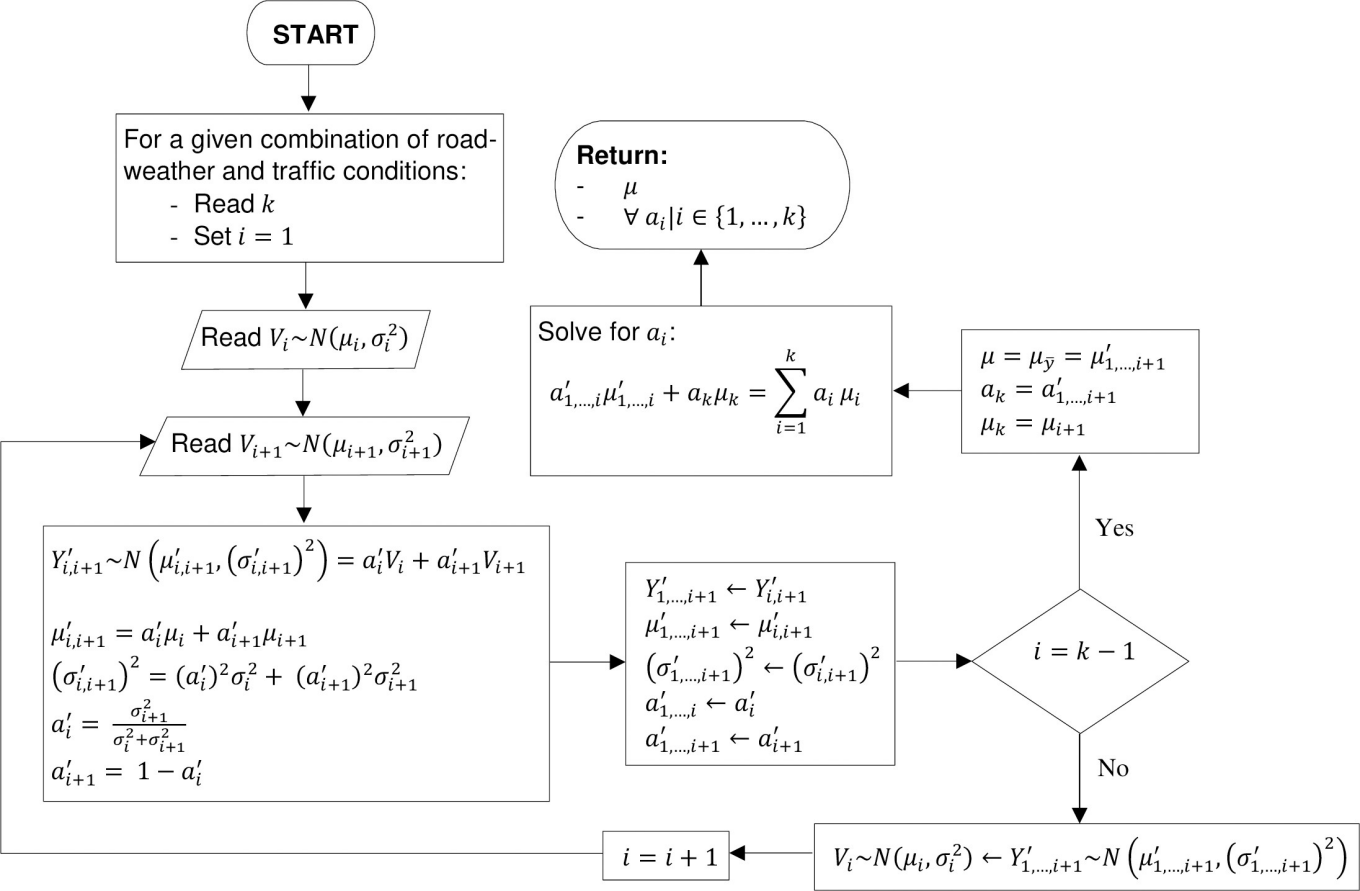
Algorithm for estimation of weight factors and *μ*.

A statistical approach to estimate $\mu_{\bar{y}}$ involves solving Eqs 2, 3 and 4 while minimizing ${\sigma_{\bar{y}}}^{2}$ which eventually yields the MVUE of $\mu_{\bar{y}}$. Nevertheless, the aforementioned system of equations consists of *k*+2 degree of freedom including $\mu_{\bar{y}},\;{\sigma_{\bar{y}}}^{2}$ and *a*_*i;i* = 1 *to k*_, with only four constraints (i.e., Eqs 2 through 4 and variance minimization equation) expressing the relationship between the aforementioned variables. Accordingly, the system of equations becomes indeterminate for *k*>2. However, the system of equations can be still resolved for *k* = 2 by treating the cluster of sampling distributions of five-minute aggregate speeds as a chain of the products of two distributions at a time. Fig 8 graphically illustrates the sequential combination procedure of *k* sampling distributions of five-minute aggregate speeds, where all *k* distributions represent an identical population. First, *k* distributions are sorted ascendingly in terms of the five-minute traffic volume (sample size). Subsequently, the first two out of *k* distributions; *V*_1_ and *V*_2_ as depicted in Fig 8, are combined linearly yielding an intermediate hypothetical distribution of five-minute aggregate speeds i.e., $Y_{1,2}^{\prime }$. Afterwards, $Y_{1,2}^{\prime }$ is chained with the third distribution of five-minute aggregate speeds *V*_3_, yielding the subsequent intermediate hypothetical distribution of five-minute aggregate speeds $Y_{1,2,3}^{\prime }$. The chaining process is continued until all *k* individual sampling distributions of five-minute aggregate speeds representing the population of individual vehicle speeds are encompassed in the intermediate hypothetical distributions, eventually producing $Y_{1,2,3,4,\ldots ,i,\ldots ,k}^{\prime }$ bearing the MVUE of $\mu_{\bar{y}}$. Accordingly, an algorithm is proposed to estimate the weight factors *a*_*i* = 1 *to k*_ and the MVUE of $\mu_{\bar{y}}$ which is equivalent to *μ* (Fig 9).

For a given combination of road-weather and traffic conditions, the algorithm presented in Fig 9 initially combines the first two individual sampling distributions of five-minute aggregate speeds; *V*_1_ and *V*_2_ according to Eqs 1, 2 and 3, such that the linear combination of *V*_1_~(*μ*_1_, *σ*_1_^2^) and *V*_2_~(*μ*_2_, *σ*_2_^2^) yields a normally distributed hypothetical distribution of five-minute aggregate speeds; $Y_{1,2}^{\prime }∼\left(\mu_{1,2}^{\prime },{\left(\sigma_{1,2}^{\prime }\right)}^{2}\right)$ possessing the MVUE of $\mu_{1,2}^{\prime }$, which corresponds to the most representative unbiased estimate of the mean of $Y_{1,2}^{\prime }$. In other words, $Y_{1,2}^{\prime }$ holds the minimum variance among all distributions representing the linear combination of *V*_1_ and *V*_2_. The proposed methodology for estimating the exact value of the MVUE of $\mu_{1,2}^{\prime }$ is presented below.

The linear combination of *V*_1_ and *V*_2_ corresponds to the bivariate linear combination of two (i.e., *k* = 2) sampling distributions of five-minute aggregate speeds. The linear combination ultimately produces $Y_{1,2}^{\prime }$ (Eq 1 with *k* = 2) which is a normally distributed intermediate random variable characterized by its mean $\mu_{1,2}^{\prime }$ and variance ${\left(\sigma_{1,2}^{\prime }\right)}^{2}$ estimated according to Eqs 2 and 3 respectively with *k* = 2. The values of $\mu_{1,2}^{\prime }$, $a_{1}^{\prime },\;a_{2}^{\prime }$ and ${\left(\sigma_{1,2}^{\prime }\right)}^{2}$ are estimated as explained below, while the coefficients in Eqs 2 and 3 i.e., $\mu_{1},\;\sigma_{1}^{2},\;\mu_{2}$ and $\sigma_{2}^{2}$ (mean and variance of sampling distributions) are known for a specific population.

The weight factors $a_{1}^{\prime }$ and $a_{2}^{\prime }$ represent the respective contribution of *V*_1_ and *V*_2_ in $Y_{1,2}^{\prime }$. Therefore, the two weight factors $a_{1}^{\prime }$ and $a_{2}^{\prime }$ sum up to 1 (Eq 4). Accordingly, the variance of $Y_{1,2}^{\prime }$ is expressed by:

$${\left(\sigma_{1,2}^{\prime }\right)}^{2}={{\left(a_{1}^{\prime }\right)}^{2}\sigma }_{1}^{2}+{\left(1-a_{1}^{\prime }\right)}^{2}\sigma_{2}^{2}$$ Eq 5

The weight factors $a_{1}^{\prime }$ and $a_{2}^{\prime }$ are estimated by setting the first derivative of ${\left(\sigma_{1,2}^{\prime }\right)}^{2}$ with respect to $a_{1}^{\prime }\;\left(i.e.\;d{\left(\sigma_{1,2}^{\prime }\right)}^{2}/da_{1}^{\prime }\right)$ to zero to obtain the minimum variance of $Y_{1,2}^{\prime }$ yielding,

$$a_{1}^{\prime }=\frac{\sigma_{2}^{2}}{{\sigma_{1}^{2}+\sigma }_{2}^{2}}$$ Eq 6

Substituting $a_{1}^{\prime }$ in Eq 4 (*k* = 2) yields:

$$a_{2}^{\prime }=\frac{\sigma_{1}^{2}}{{\sigma_{1}^{2}+\sigma }_{2}^{2}}$$ Eq 7

Substituting $a_{1}^{\prime }$ and $a_{2}^{\prime }$ in Eq 1, Eq 2 and Eq 3 (*k* = 2) yields:

$$Y_{1,2}^{\prime }=\frac{\sigma_{2}^{2}}{{\sigma_{1}^{2}+\sigma }_{2}^{2}}\;V_{1}+\frac{\sigma_{1}^{2}}{{\sigma_{1}^{2}+\sigma }_{2}^{2}}V_{2}$$ Eq 8

$$\mu_{1,2}^{\prime }=\frac{\sigma_{2}^{2}}{{\sigma_{1}^{2}+\sigma }_{2}^{2}}\mu_{1}+\frac{\sigma_{1}^{2}}{{\sigma_{1}^{2}+\sigma }_{2}^{2}}\mu_{2}$$ Eq 9

$${\left(\sigma_{1,2}^{\prime }\right)}^{2}={\left(\frac{\sigma_{2}^{2}}{{\sigma_{1}^{2}+\sigma }_{2}^{2}}\right)}^{2}\sigma_{1}^{2}+{\left(\frac{\sigma_{1}^{2}}{{\sigma_{1}^{2}+\sigma }_{2}^{2}}\right)}^{2}\sigma_{2}^{2}$$ Eq 10

$Y_{1,2}^{\prime }$ is then chained with the next sampling distribution of five-minute aggregate speeds; *V*_3_ and a new intermediate hypothetical normally distributed distribution of five-minute aggregate speeds $Y_{1,2,3}^{\prime }$ is produced. This process is continued until all *k* original individual distributions of five-minute aggregate speeds are integrated in the chain of the resulting hypothetical intermediate distributions. Consequently, the ultimate hypothetical distribution of five-minute aggregate speeds; $Y_{1,2,3,4,\ldots ,i,\ldots ,k}^{\prime }$ conveys the characteristics of the population of five-minute aggregate speeds representing the distribution of individual desired speeds observed under a unique combination of road-weather and traffic conditions. Therefore, $Y_{1,2,3,4,\ldots ,i,\ldots ,k}^{\prime }$ is equivalent to *Y* as expressed in Eq 1 and referred to as *Y* hereinafter. Moreover, *Y* possesses the minimum variance (${\sigma_{\bar{y}}}^{2}$) among all combinations of *k* sampling distributions of five-minute aggregate speeds leading *Y* to be the most representative representation of the distribution of individual speeds under a particular combination of road-weather and traffic conditions (a population). Consequently, the mean of *Y* i.e., $\mu_{\bar{y}}$ is concluded as the MVUE for the population mean, *μ*.

Estimating the MVUE of $\mu_{\bar{y}}$ according to the proposed algorithm for a population represented by three sampling distributions (*k* = 3) is elaborated in the S1 Appendix.

### Standard deviation of desired speeds’ distribution (*σ*) of individual vehicles’ population

As explained before, the linear combination of *k* sampling distributions of five-minute aggregate speeds yields to *Y* (Eq 1), which is characterized by a variance of ${\sigma_{\bar{y}}}^{2}$ (Eq 3). On the other hand, *σ*_*i*_, denotes the standard deviation of the *i*^th^ sampling distribution of five-minute aggregate speeds (*V*_*i*_) pertaining to a specific sample size (five-minute traffic volume *n*_*i*_). *V*_*i*_ is one out of *k* sampling distributions representing the population of desired speeds of individual vehicles observed under a unique combination of road-weather and traffic conditions with a standard deviation *σ*. Eq 11 defines the relationship between the standard deviation of the population (*σ*) and that of *i*^th^ sampling distribution (*σ*_*i*_):

$$\sigma_{i}=\sigma /\sqrt{n_{i}}$$ Eq 11

Substituting *σ*_*i*_ from Eq 11 to Eq 3 yields,

$${\sigma_{\bar{y}}}^{2}=a_{1}^{2}\left(\frac{\sigma^{2}}{n_{1}}\right)+\cdots +a_{i}^{2}\left(\frac{\sigma^{2}}{n_{i}}\right)+\cdots +a_{k}^{2}\left(\frac{\sigma^{2}}{n_{k}}\right)$$ Eq 12

After rearranging Eq 12, the standard deviation of the distribution of desired speeds of individual vehicles observed under a unique combination of road-weather and traffic conditions, i.e., the population standard deviation can be expressed as:

$$\sigma =\sqrt{\frac{{\sigma_{\bar{y}}}^{2}}{\sum_{i=1}^{k}a_{i}^{2}/n_{i}}}$$ Eq 13

### Identification of road-weather conditions with intensified safety risks

To investigate the impacts of adverse road-weather conditions on the desired speed distribution characteristics, the combinations of road-weather conditions under prevailing traffic conditions, i.e., traffic flow values from 100 veh/h to 200 veh/h (Fig 2) and heavy vehicle percentages from 20% and 30% (Fig 4(A)) are explored.

Safety risks in this study are evaluated in terms of two important aspects of a potential crash i.e., severity and propensity. Speed and the variability of speeds are often acknowledged as appropriate measures in evaluating crash severity and crash involvement respectively, where high speeds and high standard deviation of speeds are associated with severe injuries occurred at a potential crash and high crash involvement rates respectively [36]. A weather-related crash (hereinafter crash), however, involves the presence of adverse road-weather conditions. Moreover, combination of the most frequent road-weather conditions (dry pavement surface condition, no precipitation, daytime and temperature values between -10°C and 0°C in the context of this study) can be considered as normal road-weather conditions due to drivers’ frequent exposure to such road-weather conditions. Therefore, the severity and the propensity of a weather-related crash can be accurately evaluated by assessing the difference in the desired speed distribution characteristics under normal and adverse road-weather conditions considering prevailing traffic conditions.

The distributions of desired speeds under prevailing traffic conditions encompass two types of road-weather conditions: normal and adverse. The term “adverse” refers to any combination of road-weather conditions under prevailing traffic conditions. In this study, we denote the mean and standard deviation of a desired speed distribution belonging to prevailing traffic and normal road-weather conditions by *μ*_*Normal*_ and *σ*_*Normal*_ respectively, where the mean and standard deviation of a desired speed distribution belonging to prevailing traffic and adverse road-weather conditions are denoted respectively by; *μ*_*Adverse*_ and *σ*_*Adverse*_. We propose to express *μ*_*Adverse*_ and *σ*_*Adverse*_ as functions of *μ*_*Normal*_ and *σ*_*Normal*_ respectively, considering crash severity and exposure factors as in Eqs 14 and 15.

$$\mu_{Adverse}=\alpha_{Adverse}\times \mu_{Normal}$$ Eq 14

$$\sigma_{Adverse}=\beta_{Adverse}\times \sigma_{Normal}$$ Eq 15

where,

*α*_*Adverse*_: Crash Severity Factor (CSF)

*β*_*Adverse*_: Crash Exposure Factor (CEF)

Mean speed is well-acknowledged as an indication of crash severity [36]. Therefore, CSF is considered as a surrogate measure for crash severity under a particular combination of road-weather conditions. Considering mean desired speed under normal road-weather conditions as a reference, road-weather conditions with CSF≤1 imply lower (or equal) mean desired speed (*μ*_*Adverse*_≤*μ*_*Normal*_). Thus, potential crashes under such road-weather conditions are classified as “low severity” as compared to normal road-weather conditions. In contrast, potential crashes under road-weather conditions with CSF>1 are classified as “high severity”, due to higher mean desired speed (*μ*_*Adverse*_>*μ*_*Normal*_).

Similarly, CEF i.e., defined based on standard deviation of desired speed under a particular combination of road-weather conditions, is considered as a surrogate measure for crash propensity [37]. Road weather conditions with CEF≤1 are classified as “low exposure” due to lower variability of desired speeds (*σ*_*Adverse*_≤*σ*_*Normal*_). On the contrary, road-weather conditions with CEF>1 are identified as “high exposure” due to high standard deviations of desired speeds as compared to normal road-weather conditions (*σ*_*Adverse*_>*σ*_*Normal*_).

Subsequently, each combination of road-weather conditions with the most frequent traffic conditions are classified into four categories and labelled in terms of crash severity and exposure depending on the values of CSF and CEF as shown in Table 3.

**Table 3:** Classification criteria for combinations of road-weather conditions to identify road-weather conditions with intensified safety risks.

	CEF ≤1	CEF >1
**CSF ≤1**	Low severity, Low exposure	Low severity, High exposure
**CSF >1**	High severity, Low exposure	High severity, High exposure

Conditions labelled as “High severity, High exposure” are identified as the road-weather conditions imposing the highest safety risks followed by conditions labelled “High severity, Low exposure”, “Low severity, High exposure” and “Low severity, Low exposure” in a descending order with respect to safety risks.

## Modeling results

Data collected from the study site encompassed 933 unique combinations of road-weather (precipitation condition, pavement surface condition, time of the day, air temperature) and traffic conditions (traffic flow, heavy vehicles percentage) as shown in Fig 5. Accordingly, the desired speed distributions of each of the 933 combinations were modelled as normal distributions characterized by mean (*μ*) and standard deviation (*σ*).

**Fig 10:**
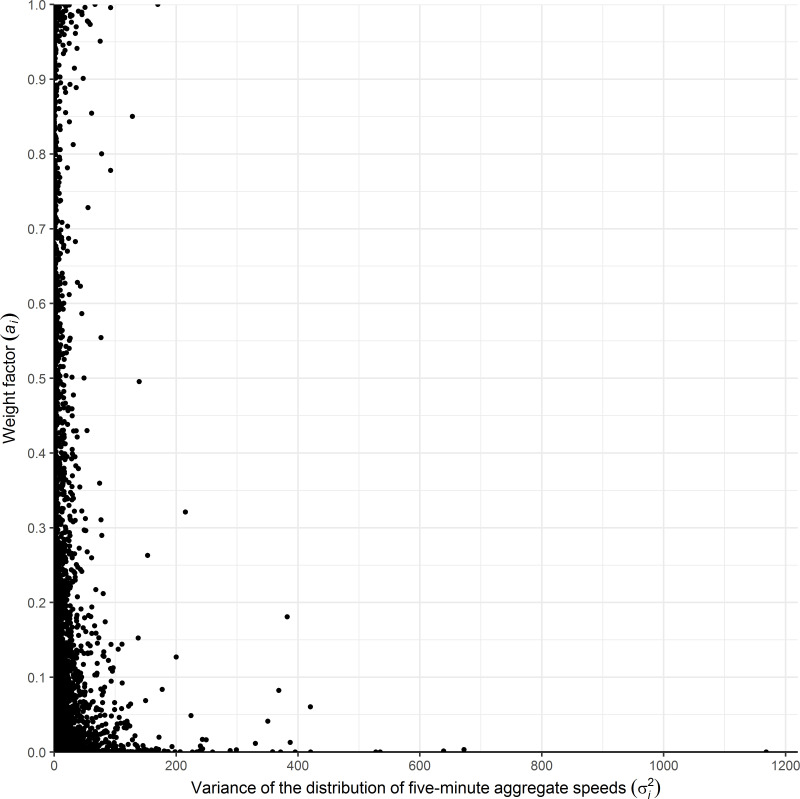
Weight factor VS the variance of the distributions of five-minute aggregate speeds.

To recall, a weight factor (*a*_*i*_) depending on the variance ($\sigma_{i}^{2}$) was assigned to each sampling distribution of five-minute aggregate speeds representing a specific desired speed population pertaining to a particular combination of road-weather and traffic conditions. With a few exceptions, the sampling distributions with a larger variance were assigned significantly smaller weight factors, i.e., contributing less toward estimating the desired speed distribution characteristics (Fig 10) that is the direct consequence of implementing MVUE. Thus, the proposed methodology assures that more emphasis is placed on sampling distributions with more stable speed observations (e.g., lower variance due to larger sample size) while preserving and using all observations.

**Fig 11:**
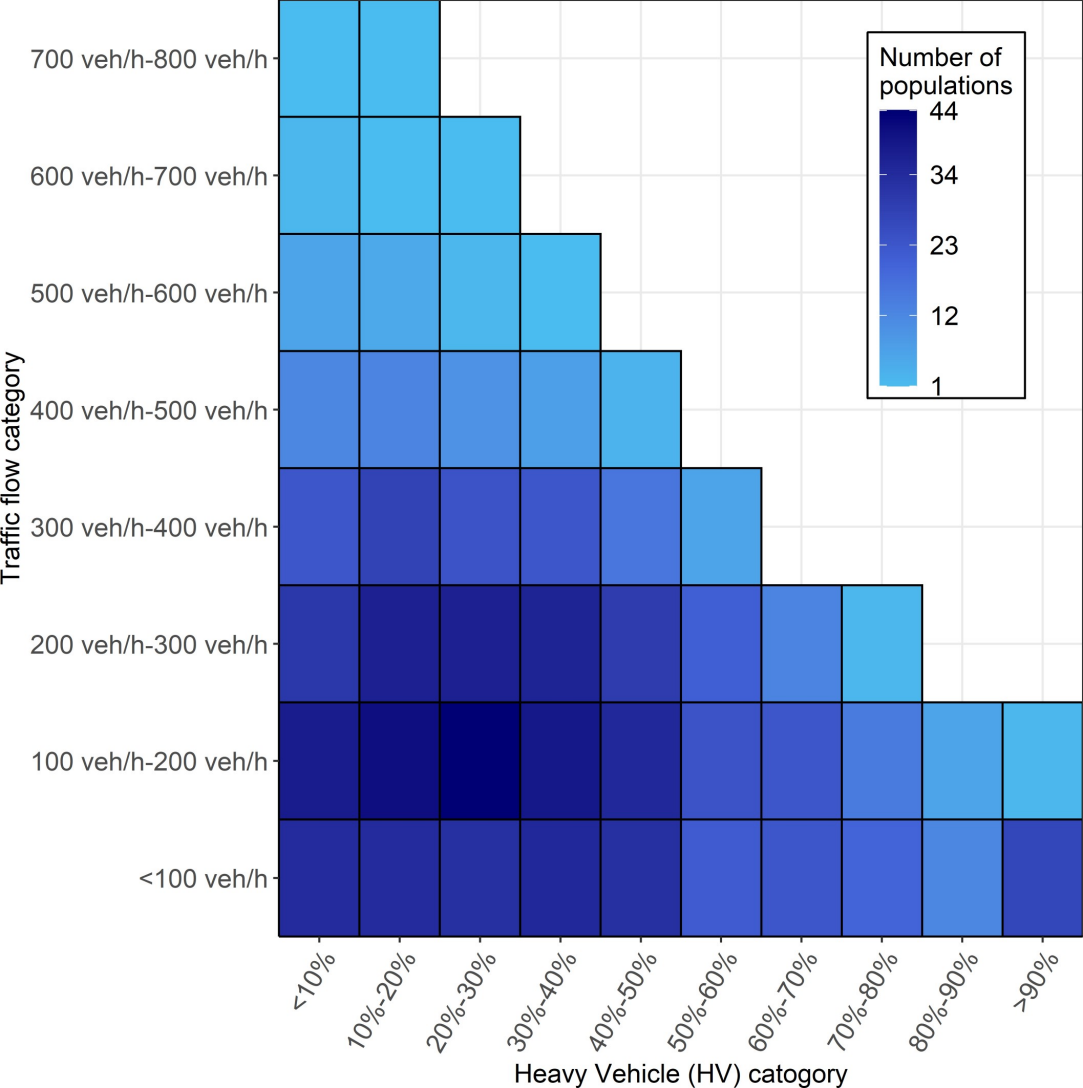
Number of populations recorded in terms of the traffic flow and HV categories.

Fig 11 presents the number of populations recorded at the study site in terms of traffic flow and Heavy Vehicle (HV) percentage conditions, indicating that 44 out of the 933 populations observed were operating under prevailing traffic conditions for which the characteristics of desired speed distributions are presented and discussed in the next section.

### Impact of road-weather and traffic conditions on estimated desired speed distributions

**Fig 12:**
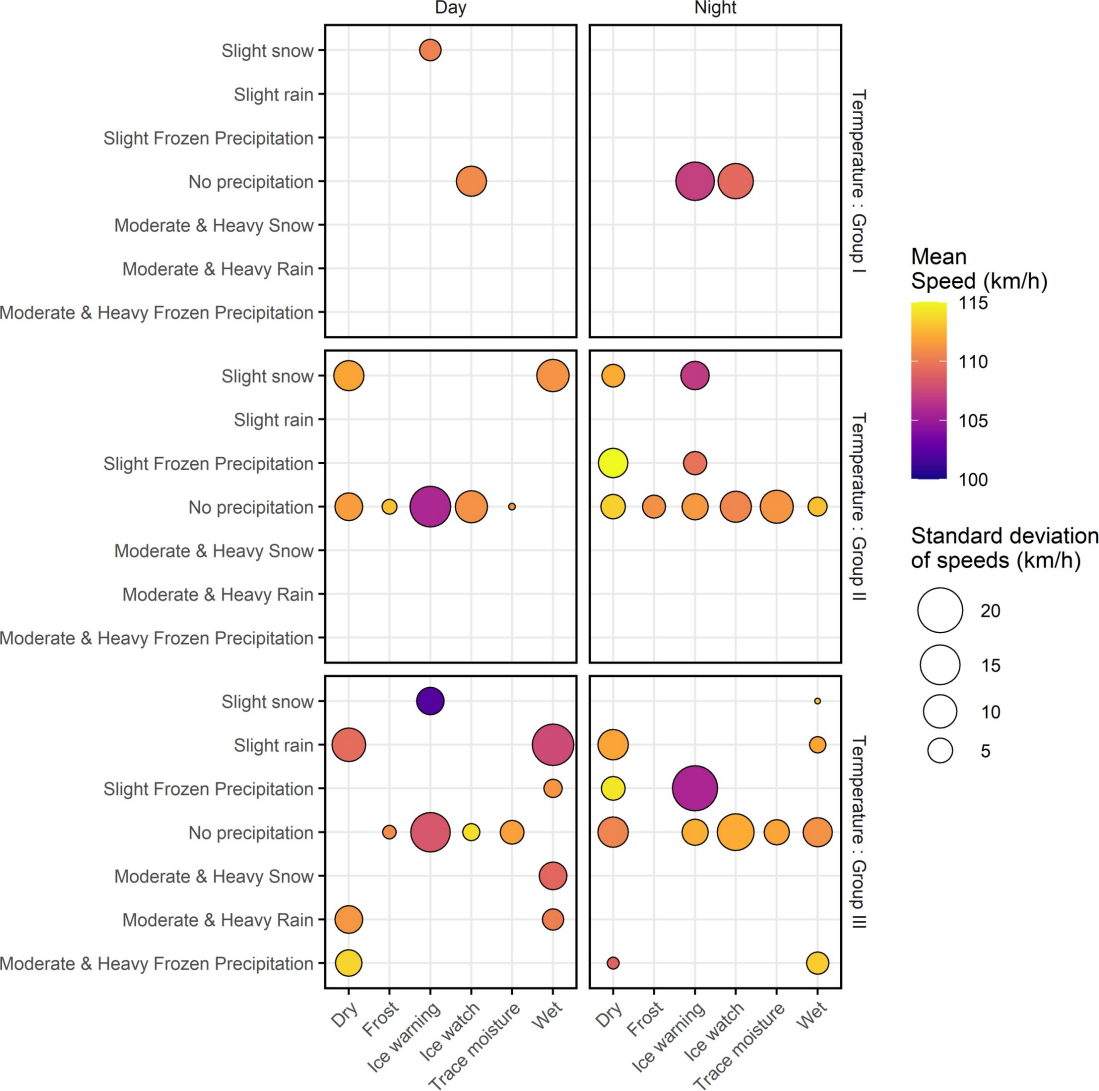
Desired speed distribution characteristics; mean (*μ*) and standard deviation (*σ*) of road-weather combinations under the prevailing traffic conditions.

Fig 12 depicts the mean (*μ*) and standard deviation (*σ*) of the desired speed distributions under 44 unique combinations of precipitation conditions, pavement surface conditions, time of the day and air temperature categories observed under prevailing traffic conditions. Distribution of desired speeds under the combination of the most frequent road-weather conditions i.e., dry pavement, no precipitation, daytime and temperature values between -10°C and 0°C (Group II) is characterized by a normal distribution with a mean of 112 km/h and a standard deviation of 6.5 km/h (Fig 12). Fig 12 reveals interesting speed behaviour idiosyncrasies. For instance, the average speed chosen by drivers travelling under ice warning pavement conditions is significantly lower than the average speed chosen by drivers travelling under other road surface conditions (dry, wet, frost, trace moisture and ice watch), irrespective of weather conditions prevailing at the time of travel. A closer inspection of Fig 12 further reveals that the desired speed distributions of vehicles travelling under ice warning road surface conditions possess comparatively higher values of standard deviation, signifying the diversified speed choices under ice warning pavement conditions. The higher numerical values for means coupled with higher standard deviation values of the desired speed distributions under ice warning road surface conditions is intuitive since majority of the drivers are very much attentive towards the perceptible roadway hazards during driving maneuvers. Nevertheless, each individual driver perceives the risk on driving on such perceptible hazards at different levels which is manifested by the high standard deviation of speeds eventually intensifying the crash risks. In general, drivers are well aware of the reduced road surface friction under icy pavement conditions and typically incline towards driving at a lower speed compared to the fixed posted speed limit of 110 km/h. The choice of such lower speed is determined by drivers predominantly based on their driving experience, confidence, and comfort levels. Accordingly, some experienced drivers may drive at higher speeds even though they are aware of the deteriorated driving conditions, leading to higher means of the desired speed distributions (e.g., under frost and or ice watch conditions) and high crash severities.

The aforesaid argument is further validated in the presence of acute precipitation. For instance, the desired speed distributions in daytime recorded with a road surface condition of ice warning, temperature values above 0°C (Group III) and slight snow evinced a phenomenally low mean with a numerical value of 102 km/h, which is the lowest mean among the 44 desired speed distributions, and a considerably low standard deviation (6.39 km/h) compared to the remaining 43 combinations of road-weather conditions (Fig 12). This observation supports the hypothesis that the majority of drivers are not comfortable driving at or near the speed limit of 110 km/h, which is considerably higher than 102 km/h. Moreover, the low standard deviation of the aforementioned desired speed distribution suggests a comparatively low variability in the desired speeds implying the alike collective judgment of a safe speed among the majority of the drivers. In contrast, the highest variability of speeds prevails under ice warning pavement, slight frozen precipitation, nighttime and temperature values above 0°C (Group III) which is manifested through a standard deviation of 20.07 km/h. Nevertheless, mean of the aforementioned desired speed distribution was estimated as 106 km/h, which is comparatively low with respect to the rest of the desired speed distribution means presented in Fig 12. This inconsistency is potentially due to the unexpected slight frozen precipitation occurring at peculiar temperature values, leading the drivers to prefer speeds in a wide range depending on their confidence and comfort of driving at a particular speed, yielding a significantly higher standard deviation of the distribution of desired speeds.

Surprisingly, however, the presence of precipitation alone does not seem to jeopardize drivers’ uncertainty towards adapting a safe speed to travel under most precipitation conditions. This is evident from the comparatively higher means of the desired speed distributions under dry pavement conditions even in the presence of frozen precipitation and snowy conditions. In fact, the highest mean desired speed (115 km/h) among the combinations of road-weather conditions considered in Fig 12 was under dry pavement, temperature values between 0°C and -10°C (Group II), nighttime and slight frozen precipitation conditions. Nevertheless, the aforementioned desired speed distribution possesses a comparatively low standard deviation (7.49 km/h) signaling a moderate homogeneity of desired speeds under particular combination of road-weather conditions. On the other hand, the combination of road-weather conditions; wet pavement, slight snow, nighttime and temperature values above 0°C (Group III) recorded the minimum standard deviation of desired speed distributions in Fig 12 with a value of 0.39 km/h. Meanwhile, the mean of the same desired speed distribution is surprisingly high with a value of 113 km/h. These rather contradictory results can be attributed to driver experience as a result of frequent exposure to driving under inclement road-weather conditions. As discussed before, the study site is located in an extremely cold region which is subjected to frequent adverse road-weather conditions. Hence, it could conceivably be hypothesized that frequent exposure to hazardous driving conditions could be a major factor in the inferred weak link between the adverse road-weather conditions and the selection of a lower speed as a safe speed to travel, which is manifested by the high mean and the extremely low standard deviation of the aforesaid desired speed distribution.

With successive increases in the intensity of the atmospheric temperature, the mean speed of the desired speed distributions gradually increases in the case of ice warning, no precipitation and nighttime conditions. Interestingly, however, both the minimum and the maximum standard deviation of desired speed distributions under prevailing traffic conditions emerged under identical time of the day and temperature group, which are nighttime and temperature values above 0°C (Group III) respectively. This is rather an important outcome as it reveals that the variability of speeds is particularly affected by the combination of precipitation and road surface condition. Nevertheless, there is no convincing evidence to conclude a significant relationship between the desired speed distribution characteristics and the two road-weather conditions, time of the day and temperature.

In summary, the characteristics of the desired speed distributions suggest that there is a strong association principally with the two road-weather conditions i.e., road surface and precipitation conditions. In particular, the speed considered safe by each driver under identical road-weather conditions seems to be dependent on their personal comfort levels and confidence to travel, specially under perilous pavement conditions which is manifested through divergent values of desired speed distribution means and standard deviations.

### Adverse road-weather conditions with intensified road safety risks

**Fig 13:**
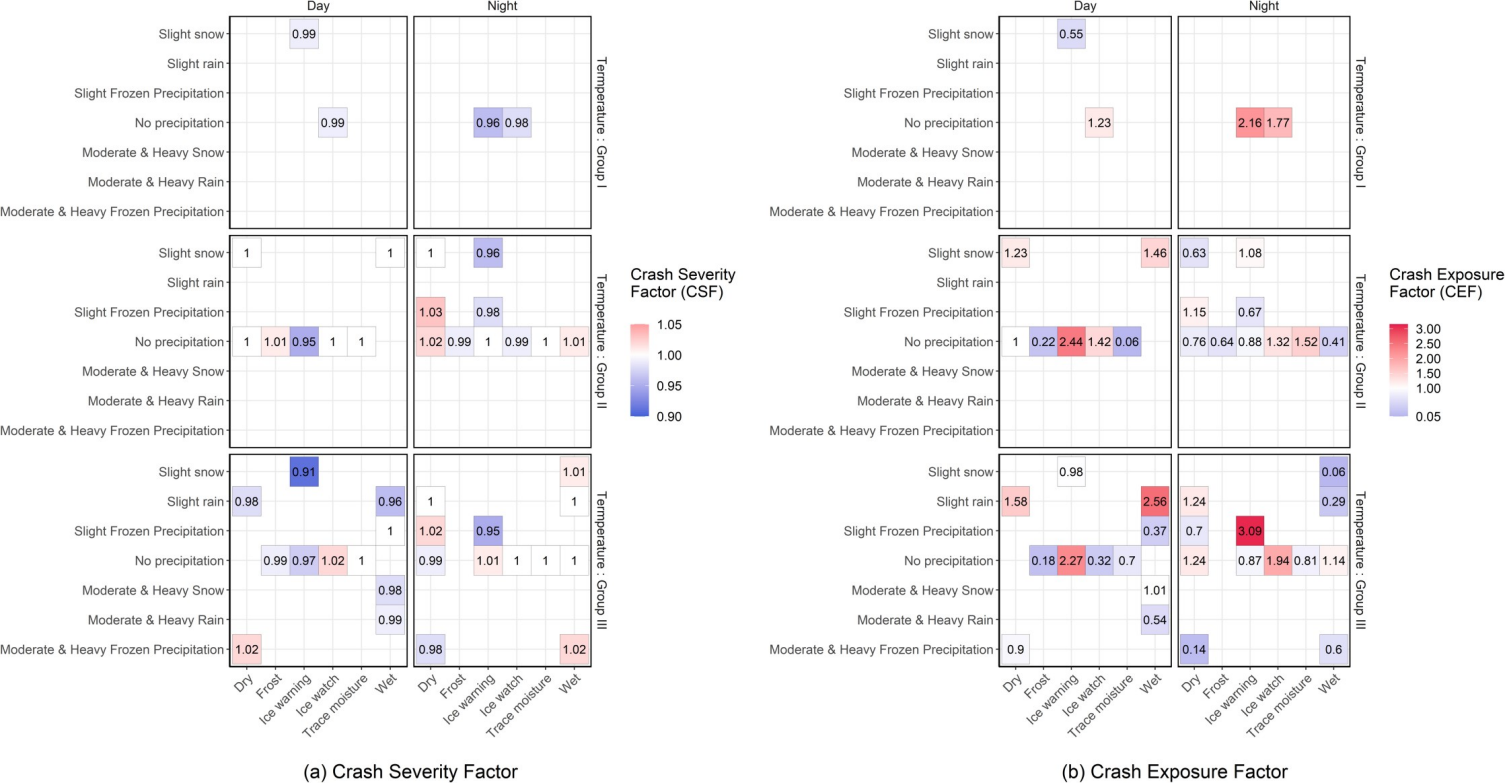
Crash severity and crash exposure factors for each road-weather combination under the prevailing traffic conditions.

To identify the specific road-weather conditions with intensified safety risks, mean and standard deviation of desired speed distribution pertaining to a normal road-weather and normal traffic conditions; *μ*_*Normal*_ and *σ*_*Normal*_ were first estimated as 112 km/h and 6.5 km/h, respectively. Consequently, CSF and CEF values estimated for each combination of road-weather conditions under prevailing traffic conditions were estimated as presented in Fig 13(A) and 13(B), respectively. The maximum CSF corresponds to the dry pavement, temperature values between 0°C and -10°C (Group II), nighttime and slight frozen precipitation conditions, while the minimum CSF corresponds to ice warning road surface conditions, temperature values above 0°C (Group III), daytime and slight snow conditions (Fig 13(A)). In terms of the CEF, the maximum crash exposure is estimated for combination of ice warning road surface conditions, temperature values above 0°C (Group III), slight frozen precipitation and nighttime. The minimum CEF is estimated for the combination of wet road surface conditions, temperature values above 0°C (Group III), slight snow and nighttime (Fig 13(B)).

**Fig 14:**
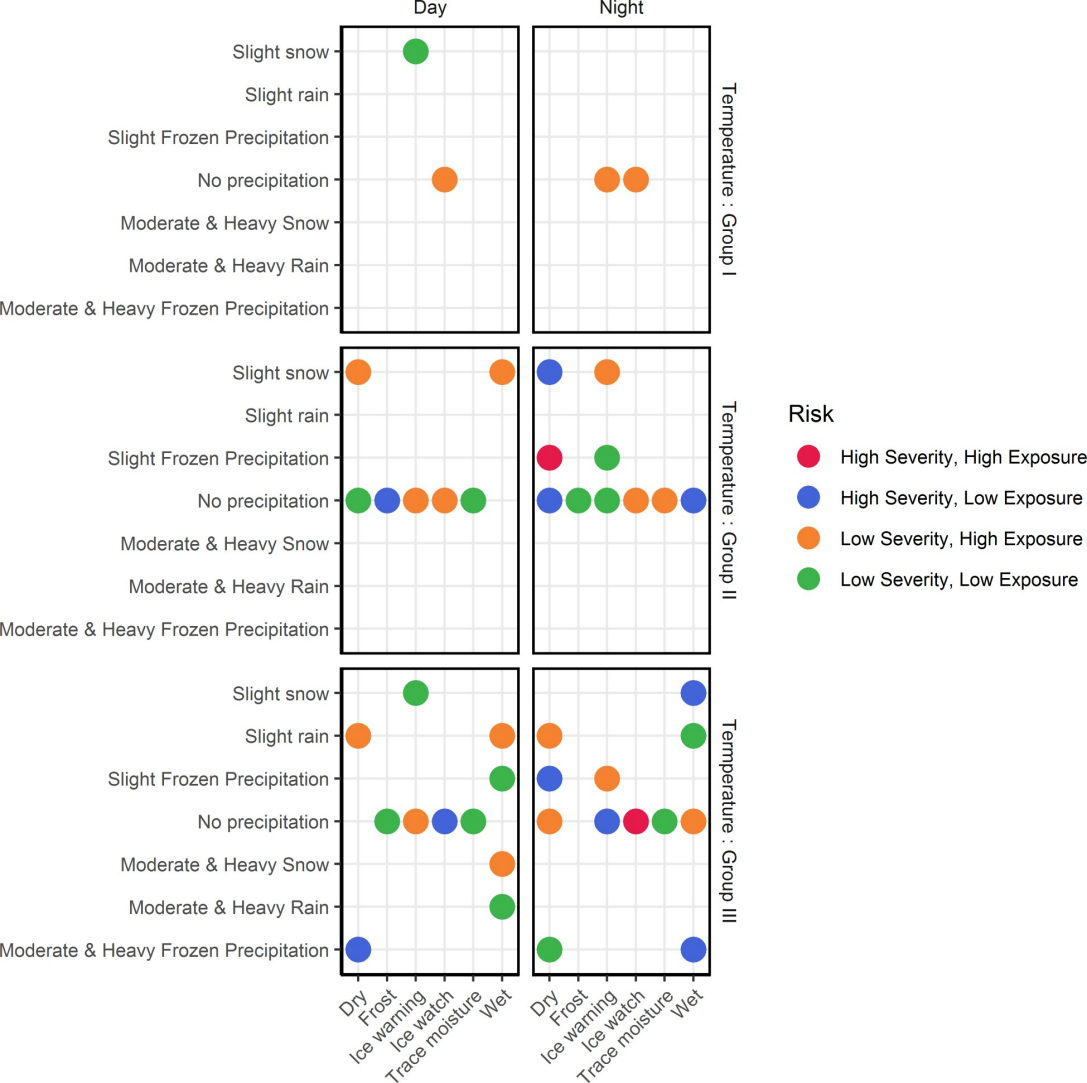
Classification of each road-weather combination under the prevailing traffic conditions.

Fig 14 classifies each combination of road-weather condition observed under prevailing traffic conditions in terms of potential safety risks as defined in Table 3. According to Fig 14, only two combinations of road-weather conditions are classified in the category of extremely high potential safety risks, i.e., the combination of *i)* ice watch road surface, temperature values above 0°C (Group III), nighttime and no precipitation conditions, and *ii*.*)* dry pavement, temperature values between 0°C and -10°C (Group II), nighttime and slight frozen precipitation conditions. Moreover, 14 out of the 44 combinations of road-weather conditions considered in the analysis were classified in the category with no significant potential safety risks compared to reference conditions while 10 combinations were classified in the category with high potential risk of severe crashes. Finally, 18 combinations were classified in the category with high potential risk of crash occurrence.

Overall, 68% of road-weather combinations under prevailing traffic conditions are classified to be hazardous in terms of either potential crash severity or potential exposure to crashes, which conveys the vulnerability of traffic safety under adverse road-weather conditions.

### Comparative analysis

To the extent of our knowledge, no study has modelled the desired speed distributions under the combination of road-weather and traffic conditions for rural highways in cold regions even though several past studies have estimated the speed variations resulted by adverse road-weather conditions [9, 12]. Nevertheless, the study results relating to the most frequent combinations of traffic conditions (i.e., traffic flow values between 100–200 veh/h and heavy vehicle percentages between 20%-30%) are compared with [9] and [11]. Yasanthi & Mehran [9] used regression modelling to investigate the same study site and data as in the current study which provides consistent basis for comparison.

Therefore, speed reduction factors for vehicles travelling in the shoulder lane under specific combinations of road-weather and traffic conditions estimated by Group II models (linear regression models with 20-minute aggregate speeds as the dependent variables) as suggested by [9] were considered for comparative evaluation. Further, speed reduction factors suggested by [11] were also considered for evaluation and comparison. It should be noted that numerical values for the temperature, heavy vehicle percentage and traffic flow are used to allow better comparison as well as to represent a practical application of the populations. For instance, a numerical value of 5°C is used for the comparison as compared to using the temperature group of “GIII” (Table 1) which enables direct estimation of the speed reduction suggested by [9] and [11].

Table 4 presents speed reduction factors (comparing to reference normal road-weather conditions) under four combinations of road-weather conditions. To allow better comparison, only slight and heavy snow are considered. The results show that desired speed distributions under different road-weather conditions can be characterized by considerably distinctive means and standard deviations which implies that the speed behaviour of drivers travelling in rural divided highways under adverse road-weather conditions is affected by pertaining road-weather conditions, which is consistent with [9] and [11] observations. Both the present study and [9] revealed that the choice of desired speed is predominantly influenced by drivers’ precipitation and perceptible road surface conditions at the time of travel.

**Table 4:** Comparison of results with past literature.

			Combination of Road-weather conditions			
			Combination I	Combination II	Combination III	Combination IV
Road-weather condition	Pavement condition		Ice warning	Wet	Wet	Ice warning
	Precipitation Intensity (mm/h)		1	1	9	1
	Temperature (°C)^1^		5	5	5	-15
	Time of the day		Daytime	Nighttime	Daytime	Daytime
Traffic condition^1^	Traffic flow (veh/h)		150 veh/h	150 veh/h	150 veh/h	150 veh/h
	Heavy vehicles percentage (%)		20%	20%	20%	20%
Desire speed distribution characteristics	Mean (Km/h)		102	113	109	110
	Standard deviation (Km/h)		6.39	0.39	6.53	3.58
Speed reduction factors^2^	Present study^3^		8.93%	-0.89%	2.68%	1.79%
	Yasanthi & Mehran (2020)	Light vehicles^4^	2.15%	2.16%	3.79%	3.73%
		Heavy vehicles^5^	4.64%	3.47%	1.67%	6.03%
	HCM (2016)^6^		13%	13%	16%	13%

^1^ Numerical values used to allow better comparison of the existing study’s results with [9] and [11].

^2^$Speed\;reduction\;factor=\frac{\left(Speed\;under\;normal\;road-weather)-(Speed\;under\;adverse\;road-weather\right)}{Speed\;under\;normal\;road-weather}\times 100$.

^3^ Speed under normal road-weather conditions: 112 km/h.

^4^ Speed under normal road-weather conditions: 117 km/h.

^5^ Speed under normal road-weather conditions: 116 km/h.

^6^ Speed reduction factors based on HCM (2016) only based on the precipitation condition and a base free-flow speed of 113km/h (70 mi/h in Exhibit 11–21).

First, it should be noted that the speed reduction factors (Table 4) estimated by Yasanthi & Mehran [9], HCM [11] and the current study are founded upon fundamentally different approaches, which in turn lead to different speed reduction factors as anticipated. For instance, [9] inferred free-flow speed reductions under different road-weather conditions based on linear regression models while [16] proposed free-flow speed reductions under different precipitation conditions based on stepwise regression models developed by [38]. Yet, as explained earlier, representing speed behaviour under adverse road-weather conditions through regression models is challenging especially considering restricted sampling conditions occurring under adverse road-weather conditions. For example, each individual 20-minute aggregate speed used in the “Group II” models in [9] equally contribute to estimating the regression coefficients irrespective of the number of vehicles (sample size) observed in the respective 20-minute intervals. Therefore, the suitability of a sample-level analysis such as regression modelling to study the impacts of adverse road-weather conditions on desired speed is questionable. In contrast, the current study results are based on a population-level analysis that differentiates the contribution of each five-minute aggregate speed observed in a particular combination of road-weather conditions upon the variance of the distributions of five-minute aggregate speeds representing a specific population. Yasanthi & Mehran [9] concluded one of the linear regression models produced in their study as the best performing model to represent the relationship between free-flow speed and road-weather conditions. Yet, the dependent variable of the regression models (free-flow speed) only provides an average estimation of the free-flow speed under specific adverse road-weather conditions unlike the current study, which estimates the distribution of all possible desired speeds under specific road-weather conditions. Further, the regression models developed by [9] include some statistically insignificant regression coefficients pertaining to certain road-weather conditions implying the absence of a linear relationship between such road-weather conditions and desired speed. In contrast, the present study only uses four road-weather conditions with statistically different levels for each road-weather condition (Table 1). Moreover, it should be noted that the speed reduction factors estimated according to [9, 11] and the present study (Table 4) respectively consider six, one and four road-weather conditions leading the speed reductions estimated by the three studies to spread among a different number of factors.

The speed reduction factors suggested in [11] are based on road-weather data collected from an automated surface observing system (ASOS) located in nearby airports as compared to the alongside data collection devices used in both the present study and [9]. In fact [38], the underlying source for the speed reduction factors proposed in [11], highlighted the issue of obtaining highly representative microclimate data to represent the prevailing road-weather conditions at the traffic counters. Besides, [11] estimates correspond to a different geographical region possessing unique characteristics of the driver population, resulting considerably divergent speed reduction factors compared to the present study.

## Conclusion and future directions

This study proposed a novel approach based on Central Limit Theorem to model desired speed distributions (mean (*μ*) and standard deviation (*σ*)) of vehicles travelling in rural divided highways under different combinations of road-weather and traffic conditions, followed by identification of the combinations of road-weather conditions imposing significant safety risks under prevailing traffic conditions. Often, the impacts of adverse road-weather conditions on the speed behaviour are evaluated largely by enumerating the absolute speed reductions under adverse-road weather conditions estimated largely through regression analysis at a sample-level. Speed behaviour, however, reflects the intrinsically divergent driver psychology. This study, therefore, proposed a robust methodology to model the desired speed distributions at a population-level, which can in turn be used to identify road-weather and traffic conditions with potential safety risks. The theoretical models are calibrated with road-weather and traffic data collected from a study site in Alberta, Canada where a fixed speed limit of 110 km/h is implemented irrespective of the prevailing road-weather. Yet, the proposed methodology is adaptable for uncongested divided highways in other geographical locations with similar road-weather conditions.

The outcomes of the study highlight the importance of paying special attention to traffic safety under the combination of precipitation and atypical road surface conditions while the impacts of temperature and time of day were deemed insignificant. The study identified two specific combinations of road-weather conditions potentially imposing higher crash severity and involvement risks: *i*) ice watch pavements, temperature values of above 0°C, nighttime and no precipitation conditions and *ii*) dry pavements, temperature values between 0°C and -10°C, nighttime and slight frozen precipitation conditions.

To the best of our knowledge, this study is the first comprehensive attempt in proposing a coherent methodology to model the desired speed distributions under different road-weather and traffic conditions for rural divided highways located in cold regions. The study’s contributions are twofold. First, it evaluates the impacts of different road-weather and traffic conditions on the desired speed distributions in uncongested rural highways while identifying road-weather conditions with potentially higher safety risks. Second, a methodological contribution is made by proposing an innovative approach to model desired speed distributions which can be used under different sampling rates and conditions. Thus, the research findings contribute toward understanding the divergent speed behaviour which is often critiqued as a task not trivial due to *i)* versatile nature of driver psychology and *ii)* limited sample sizes observed under adverse road-weather conditions. Transportation authorities which experience extreme road-weather conditions may adapt the methodology to understand the speed behaviour to identify prominent road-weather and traffic conditions needing urgent safety precautions such as implementing a reliable weather-responsive variable speed limit under potentially high-risk road-weather and traffic conditions. The desired speed distributions can be further used as input driver behavior parameters in defining the speed distributions in microsimulation applications to realistically simulate traffic operations under different traffic and road-weather conditions.

The generalization of these results, however, is subject to certain limitations. First, the proposed methodology is only applicable under uncongested traffic conditions. Second, the methodology is not applicable for study sites with special features such as atypical road geometry and extremely heterogeneous traffic, which may violate the assumption of a normally distributed desired speed distribution. Notwithstanding these limitations, the results of this research support the idea that drivers select different speeds under different road-weather conditions depending on their attitude about a safe speed to travel irrespective of the posted speed limit. This is particularly manifested through the considerably high standard deviations of the desired speed distributions in the study area estimated for particular road-weather conditions. In other words, higher variability of speeds caused by lack of proper communication about the safe speed to travel, imposes significant safety risks by paving the way toward elevated crash involvement. Consequently, the research findings provide the following insights for future research: *i)* How reliable is the existing speed limit in terms of consistent communication of a safe speed in different road-weather conditions? *ii)* How to propose a reliable speed limit to be implemented under the combinations of road-weather and traffic conditions which are characterized by potentially higher safety risks? and *iii)* How to predict the performance of a specific speed limit prior to applying it? Accordingly, further research focusing on proposing a robust methodology to develop a reliable weather-responsive variable speed limit system which can be effectively used in uncongested rural highways in extremely cold regions are recommended.

## Supporting information

S1 Appendix Estimating the mean (μ) and the set of weight factors representing the contribution of each five-minute aggregate speed distribution in the desired speed distribution. (DOCX)
