# Supplementary material for: Modelling speed behaviour in rural highways: Safety analysis of driving under adverse road-weather conditions
Source: PLoS One. 2021 Aug 16;16(8):e0256322. doi: 10.1371/journal.pone.0256322 (PMC8367011; doi:10.1371/journal.pone.0256322)
Supplement: S1 Appendix — (DOCX) [file pone.0256322.s001.docx]

## S1 Appendix. Estimating the mean ($\boldsymbol{\mu}$) and the set of weight factors representing the contribution of each five-minute aggregate speed distribution in the desired speed distribution

A detailed demonstration of the algorithm proposed in Fig 9 where a particular desired speed distribution is represented by three five-minute aggregate speed distributions, is presented below. The algorithm presented in Fig 9, takes the parameters of the five-minute aggregate speed distributions $V_{i;i=1 to k}$ as inputs for a given road-weather and traffic conditions. The algorithm outputs the MVUE of the mean of the desired speed distribution $\mu$, resulting from the linear combination of the five-minute aggregate speed distributions $V_{i;i=1 to k}$ along with weight factors $a_{i;i=1 to k}$ representing the contribution of each five-minute aggregate speed distribution in the population-level desired speed distribution.

## Estimating the mean of the desired speed distribution ($\boldsymbol{\mu}$)

Let a specific desired speed distribution under a particular combination of road-weather and traffic conditions (i.e., a population distribution) characterized by a mean of $\mu$ and a standard deviation of $\sigma$ be represented by three (*k*=3 in Eq. 1) distinct distributions of five-minute aggregate speeds (i.e., sampling distributions). The value of *k* is read first (*k*=3 in this case), while an integer variable *i* is defined and initialized to 1 afterwards.

Thereafter, the input data; the five-minute aggregate speed distributions $V_{i}\sim N\left( \mu_{i}, \sigma_{i}^{2} \right)$ representing the desired speed distribution in question, are read and subjected to a chaining process where each iteration of the chaining process linearly combines at least one of the five-minute aggregate speed distributions with either another five-minute aggregate speed distribution or an intermediate hypothetical five-minute aggregate speed distribution. As stated earlier, the chaining process is initiated with *i=* “1” yielding,

| ${V_{i}=V}_{1}\sim N\left( \mu_{1},{\sigma_{1}}^{2} \right)$ | Eq. 16 |
| --- | --- |

And

| $V_{i+1}=V_{2}\sim N\left( \mu_{2},{\sigma_{2}}^{2} \right)$ | Eq. 17 |
| --- | --- |

Linear combination of $V_{1}$ and $V_{2}$ yields the first intermediate hypothetical normal distribution $Y_{1,2}^{'}$, representing the weighted combination of $V_{1}$ and $V_{2}$, expressed by:

| $Y_{i,i+1}^{'}=Y_{1,2}^{'}=a_{1}^{'}V_{1}+a_{2}^{'}V_{2}\sim N\left( \mu_{1,2}^{'},\left( \sigma_{1,2}^{'} \right)^{2} \right)$ | Eq. 18 |
| --- | --- |
| $\mu_{i,i+1}^{'}=\mu_{1,2}^{'}=a_{1}^{'}\mu_{1}+a_{2}^{'}\mu_{2}$ | Eq. 19 |
| $\left( \sigma_{i,i+1}^{'} \right)^{2}=\left( \sigma_{1,2}^{'} \right)^{2}=\left( a_{1}^{'} \right)^{2}\sigma_{1}^{2}+\left( a_{2}^{'} \right)^{2}\sigma_{2}^{2}$ | Eq. 20 |

The weight factors $a_{1}^{'}$ and $a_{2}^{'}$respectively, represent the contribution of the five-minute aggregate speed distributions $V_{1}$ and $V_{2}$ in the resulting hypothetical five-minute aggregate speed distribution $Y_{1,2}^{'}$. Thus, the weight factors $a_{1}^{'}$ and $a_{2}^{'}$ can be estimated by minimizing the variance of $Y_{1,2}^{'}$ yielding:

| $a_{1}^{'}=\frac{\sigma_{2}^{2}}{\sigma_{1}^{2}+\sigma_{2}^{2}}$ | Eq. 21 |
| --- | --- |

and

| $a_{2}^{'}=1-a_{1}^{'}$ | Eq. 22 |
| --- | --- |

Subsequently, the parameters of the first intermediate hypothetical five-minute aggregate speed distribution i.e., $Y_{1,2}^{'}$ and the weight factors $a_{1}^{'}$ and $a_{2}^{'}$which are respectively associated with the linear combination of $V_{1}$ and $V_{2}$ are stored before proceeding to the next step of the algorithm. The intermediated hypothetical five-minute aggregate speed distribution $Y_{1,2}^{'}$ is chained with the next five-minute aggregate speed distribution until all *k* five-minute aggregate speed distributions are included in the chaining process. Accordingly, the need for the continuation of the chaining process is checked by evaluating the equality of the latest value of *i*. At the end of the first iteration, the value of *i* is equal to 1 and the value of “*k*-1” is equal to 2, leading to *i*$\neq$*k*-1, which prompts the execution of the second iteration of the chaining process. In the second iteration, the first intermediate hypothetical five-minute aggregate speed distribution $Y_{1,2}^{'}$ is linearly combined with the third five-minute aggregate speed distribution. Accordingly, variables *i* and $V_{i}$ are assigned with new values as presented below.

| $V_{i}\sim N\left( \mu_{i}, \sigma_{i}^{2} \right)\leftarrow Y_{1,2}^{'}\sim N\left( \mu_{1,2}^{'}, \left( \sigma_{1,2}^{'} \right)^{2} \right)$ | Eq. 23 |
| --- | --- |
| $i=2$ | Eq. 24 |

Subsequently, $V_{3}$ is read as the first step of the second iteration. The second iteration produces the second intermediate hypothetical five-minute aggregate speed distribution $Y_{1,2,3}^{'}$, representing the weighted combination of $Y_{1,2}^{'}$ and $V_{3}$, which is expressed by:

| $Y_{1,\ldots,i+1}^{'}=Y_{1,2,3}^{'}=a_{1,2}^{'}Y_{1,2}^{'}+a_{3}^{'}V_{3}\sim N\left( \mu_{1,2,3}^{'},\left( \sigma_{1,2,3}^{'} \right)^{2} \right)$ | Eq. 25 |
| --- | --- |
| $\mu_{1,\ldots,i+1}^{'}=\mu_{1,2,3}^{'}=a_{1,2}^{'}\mu_{1,2}^{'}+a_{3}^{'}\mu_{3}$ | Eq. 26 |
| $\left( \sigma_{1,\ldots,i+1}^{'} \right)^{2}=\left( \sigma_{1,2,3}^{'} \right)^{2}=\left( a_{1,2}^{'} \right)^{2}\left( \sigma_{1,2}^{'} \right)^{2}+\left( a_{3}^{'} \right)^{2}\sigma_{3}^{2}$ | Eq. 27 |

The weight factors $a_{1,2}^{'}$ and $a_{3}^{'}$respectively represent the contribution of the five-minute aggregate speed distributions $Y_{1,2}^{'}$ and $V_{3}$ in the resulting hypothetical five-minute aggregate speed distribution $Y_{1,2,3}^{'}$ bearing the minimum variance among the potential combinations of $Y_{1,2}^{'}$ and $V_{3}$. Thus, the weight factors $a_{1,2}^{'}$ and $a_{3}^{'}$ can be estimated by minimizing the variance of $Y_{1,2,3}^{'}$ yielding:

| $a_{1,2}^{'}=\frac{\sigma_{3}^{2}}{\left( \sigma_{1,2}^{'} \right)^{2}+\sigma_{3}^{2}}$ | Eq. 28 |
| --- | --- |

and

| $a_{3}^{'}=\frac{\left( \sigma_{1,2}^{'} \right)^{2}}{\left( \sigma_{1,2}^{'} \right)^{2}+\sigma_{2}^{2}}$ | Eq. 29 |
| --- | --- |

Like the first iteration, the values of the parameters $Y_{1,2,3}^{'}$, $a_{1,2}^{'}$ and $a_{3}^{'}$ are stored prior to proceeding to the next step in the algorithm. For a desired speed distribution represented by three distributions of five-minutes aggregate speeds, i.e., *k*=3, the chaining process is completed at the end of the second iteration as *i*=*k*-1=2 upon the completion of the second iteration of the chaining process. Therefore, the chaining process is terminated, and the intermediate hypothetical five-minute aggregate speed distribution produced at the end of the second iteration is concluded as the mean of the random variable *Y*; $\mu_{\bar{y}}$ (Eq. 2) representing the linear combination of the three five-minute aggregate distributions $V_{i;i=1 to 3}$. Accordingly, the MVUE of $\mu_{\bar{y}}$ can be expressed in terms of the final intermediate hypothetical five-minute aggregate speed distribution (Eq. 30), which is $\mu_{1,2,3}^{'}$. As explained earlier, the MVUE of $\mu_{\bar{y}}$ is also recognized as the mean of the population mean $\mu$. Therefore,

| $\mu=\mu_{\bar{y}}=\mu_{1,2,3}^{'}$ | Eq. 30 |
| --- | --- |

## Estimating the set of weight factors representing the contribution of each five-minute aggregate speed distribution in estimating the desired speed distribution

Mean of the random variable *Y*; $\mu_{\bar{y}}$ for a case of *k*=3 in Eq.2 can be expressed as:

| $\mu_{\bar{y}}=\sum_{j=1}^{3} a_{j}\mu_{j}$ | Eq. 31 |
| --- | --- |

On the other hand,$\mu_{\bar{y}}$ can be also expressed in terms of $\mu_{1,2,3}^{'}$ (Eq. 30) where $\mu_{1,2,3}^{'}$ is primarily equivalent to the linear combination of $\mu_{1,2}^{'}$ and $\mu_{3}$ as expressed in Eq. 26. Similarly, $\mu_{1,2}^{'}$ (Eq. 19) is equivalent to the linear combination of $\mu_{1}$ and $\mu_{2}$. It should be noted that the weight factors $a_{i;i=1 to k}$ are yet to be estimated even though the terms $a_{1,2}^{'}, \mu_{1,2}^{'}$ and $a_{3}^{'}$ are known at this stage of the algorithm, as illustrated in Fig 9. The weight factor $a_{i}$ representing the contribution of the mean of the five-minute aggregate speed distribution $V_{i}$ in estimating $\mu_{\bar{y}}$ can be estimated by considering the coefficient of $\mu_{i}$ in Eq. 31. Further, Eq. 32 is derived by combining Eq. 26, 30 and 31:

| $a_{1,2}^{'}\mu_{1,2}^{'}+a_{3}^{'}\mu_{3}=\sum_{j=1}^{3} a_{j}\mu_{j}$ | Eq. 32 |
| --- | --- |

While Eq. 32 contains the terms $\mu_{1,2}^{'}$ and $\mu_{3}$, it does not include the terms $\mu_{1}$ and $\mu_{2}$ restricting the estimation of the weight factors $a_{1}$ and $a_{2}$. Therefore, Eq. 32 is transformed to include the terms $\mu_{1}$, $\mu_{2}$ and $\mu_{3}$. By substituting $\mu_{1,2}^{'}$ from Eq. 19 in Eq. 32:

| $a_{1,2}^{'}(a_{1}^{'}\mu_{1}+a_{2}^{'}\mu_{2})+a_{3}^{'}\mu_{3}=\sum_{j=1}^{3} a_{j}\mu_{j}$ | Eq. 33 |
| --- | --- |

Expanding the Right-Hand Side (R.H.S.) and rearranging Eq. 33 yields:

| $(a_{1}^{'}a_{1,2}^{'}{-a_{1})\mu}_{1}+(a_{2}^{'}a_{1,2}^{'}{-a_{2})\mu}_{2}+{(a}_{3}^{'}-a_{3})\mu_{3}=0$ | Eq. 34 |
| --- | --- |

Eventually, the weight factors $a_{1},a_{2}$ and $a_{3}$ can be estimated by evaluating the coefficients of the terms $\mu_{1},\mu_{2}$ and $\mu_{3}$ in Eq. 34 as presented below.

By considering the coefficient of $\mu_{1}$:

| $a_{1}^{'}a_{1,2}^{'}-a_{1}=0$ | Eq. 35 |
| --- | --- |
| $a_{1}=a_{1,2}^{'}a_{1}^{'}$ | Eq. 36 |

Substituting for $a_{1}^{'}$ and $a_{1,2}^{'}$ respectively from Eq. 21 and Eq. 28 in Eq. 36 yields:

| $a_{1}=\frac{\sigma_{2}^{2}\times\sigma_{3}^{2}}{\left( \sigma_{1}^{2}+\sigma_{2}^{2} \right)\times\left( \left( \sigma_{1,2}^{'} \right)^{2}+\sigma_{3}^{2} \right)}$ | Eq. 37 |
| --- | --- |

By considering the coefficient of $\mu_{2}$:

| $a_{2}^{'}a_{1,2}^{'}-a_{2}=0$ | Eq. 38 |
| --- | --- |
| $a_{2}=a_{2}^{'}a_{1,2}^{'}$ | Eq. 39 |

Substituting for $a_{2}^{'}$ and $a_{1,2}^{'}$ respectively from Eq. 22 and Eq. 28 in Eq. 39 yields:

| $a_{2}=\frac{\sigma_{1}^{2}\times\sigma_{3}^{2}}{\left( \sigma_{1}^{2}+\sigma_{2}^{2} \right)\times\left( \left( \sigma_{1,2}^{'} \right)^{2}+\sigma_{3}^{2} \right)}$ | Eq. 40 |
| --- | --- |

By considering the coefficient of $\mu_{3}$:

| $a_{3}^{'}-a_{3}=0$ | Eq. 41 |
| --- | --- |
| $a_{3}=a_{3}^{'}$ | Eq. 42 |

Substituting for $a_{3}^{'}$ from Eq. 29 in Eq. 42 yields:

| $a_{3}=\frac{\left( \sigma_{1,2}^{'} \right)^{2}}{\left( \sigma_{1,2}^{'} \right)^{2}+\sigma_{2}^{2}}$ | Eq. 43 |
| --- | --- |

Ultimately, the algorithm returns the values of $\mu_{1,2,3}^{'}$ as the mean of the desired speed distribution (i.e., the population) represented by the five-minute aggregate speed distributions $V_{i;i=1 to 3}$ as well as the weight factors $a_{i;i=1 to 3}$ representing the contribution of each five-minute aggregate speed distribution in the population’s desired speed distribution.
